# Medial and lateral vestibulospinal projections to the cervical spinal cord of the squirrel monkey

**DOI:** 10.3389/fneur.2024.1513132

**Published:** 2025-01-03

**Authors:** Richard Boyle

**Affiliations:** Oregon Hearing Research Center, Oregon Health & Science University, Portland, OR, United States

**Keywords:** reflex control, intracellular recording, intracellular labeling, orthodromic and antidromic identification, motoneuron innervation, vestibuloocular collic neuron

## Abstract

**Introduction:**

The brainstem vestibular nuclei neurons receive synaptic inputs from inner ear acceleration-sensing hair cells, cerebellar output neurons, and ascending signals from spinal proprioceptive-related neurons. The lateral (LVST) and medial (MVST) vestibulospinal (VS) tracts convey their coded signals to the spinal circuits to rapidly counter externally imposed perturbations to facilitate stability and provide a framework for self-generated head movements.

**Methods:**

The present study describes the morphological characteristics of intraaxonally recorded and labeled VS neurons monosynaptically connected to the 8th nerve. The visualization of axon location in the descending medial longitudinal fasciculus (MLF) differentiated ipsi- (i) and contralateral (c)-projecting MVST neurons. Vestibuloocular collic (VOC) neurons were comparably typed as cMVST cells but were also antidromically activated from the rostral MLF. Cervical-only LVST neurons projected ipsilaterally in the lateral to ventrolateral funiculi. Targets of VS axons, such as central cervical nucleus neurons, sternocleidomastoid, trapezius, and splenius motoneurons, were identified using anti- and orthodromic electrical stimuli and intra-somatically labeled to describe their local spinal morphology.

**Results:**

Thirty-five VS neurons (26% of the 134 attempted samples) were successfully labeled to permit a moderate to (near) complete reconstruction of their trajectories and synaptic innervations. VOC neurons exhibited a prolific innervation of caudal brainstem nuclei, extensively innervated laminae VII and VIII, and, to a lesser extent, lateral and ventromedial lamina IX, from C1 to C8, and on average issued 15 branches along their trajectory with 92 terminal and *en passant* boutons per branch. The VOC innervation was either uniformly distributed among the cervical segments, indicating a more global control of head and neck movement, or restricted specific spinal segments, indicating a more precise motor control strategy. The innervation pattern of iMVST axons resembled that of VOC and cMVST axons but was less extensive and supplied mostly the upper two cervical segments. LVST and cMVST neurons exhibited a predominantly equally weighted innervation of separate and joint moto- and inter-neuronal spinal circuits along their cervical trajectory.

**Discussion:**

Their extensive axon branching distribution in the ventral horn provides a redundant and variable synaptic input to spinal cell groups. This suggests a common and site-specific control of the head and neck reflexes.

## Introduction

The vestibulospinal (VS) neurons of the brainstem vestibular nuclei provide the sensory signals defining the instantaneous status of the head in space to the spinal motor circuits that control reflex head/neck and limb movements and posture. The head in space might be stationary, either in its normal posture, rotated on the neck or tilted with respect to the orientation of gravity, or moving, either during voluntary activity or involuntarily, as the result of external perturbations. The key sensory signals driving the reflex action originate from hair cells in the inner ear vestibular structures: the otolith organs, namely the utricle and the saccule, that sense the sum of inertial acceleration and orientation of the head with respect to gravity and the three orthogonally arranged semicircular canals that transduce angular head rotation [reviewed by Wilson and Melvill Jones (1)]. These continuous vestibular inputs are combined with motion cues derived from neck proprioceptors and other modalities onto the VS neurons. This central representation of the head and body in space drives the rapid reflex adjustments necessary to maintain the animal’s equilibrium and stability. These sensory-to-motor signals carried in the VS pathways interact on their spinal target neurons with involuntary motor-to-sensory signals arising from the effectors, like the position and movement of the head and neck on the trunk, and purposeful voluntary commands from cognitive centers to enable the appropriate behavioral response(s) (2). At the same time, the VS neurons provide a continuous excitatory drive, called “tonus labyrinth” (3), to the spinal motor circuits to hasten their reactive responses to external perturbations. The tonic excitation raises the resting voltage level of the neuron so that new synaptic currents imparted by descending inputs, such as from VS terminals, readily bring the neuron closer to the discharge threshold (4).

The principal VS pathways are (1) the medial vestibulospinal tract (MVST) that descends in the medial longitudinal fasciculi (MLF) and ventromedial funiculi either ipsi- or contralaterally with respect to their vestibular (8th cranial) afferent nerve input and innervates spinal neurons located mainly in the ventral horn throughout the cervical segments (5); and (2) the lateral vestibulospinal tract (LVST) that course ipsilaterally with respect to their 8th nerve input in the lateral to ventrolateral funiculi and are distinguished by two divisions: (i) a cervical-projecting tract that can target the upper cervical segments or the lower cervical segments that contributes to reflex control of shoulder and forelimb (arm) muscles or both; and (ii) a lumbosacral-projecting tract that provides a rapid synaptic drive to maintain stable posture and reflex control of the lower body.

When our head is perturbed, say for example during locomotion, signals arising in the vestibular hair cells and neck proprioceptors are generated to produce the vestibulocollic reflex (VCR) to provide head stability in space, the vestibulo-ocular reflex (VOR) to hold images of stationary objects steady on the retina (6, 7), and to a lesser extent the stretch reflex-like cervicoocular reflex (5, 8). The VCR interconnects the vestibular-nerve afferents supplying the sensory hair cells, the secondary MVST and cervical-projecting LVST neurons, and the cervical motoneurons (5, 9, 10). Another possible link of the VCR is made by vestibuloocular collic (VOC) neurons, which have dual destinations: one to the cervical spinal cord via the descending MLF, like contralateral-projecting MVST axons, and the other to the contralateral oculomotor nuclei via the ascending MLF (11–14). In addition to direct VCR pathways, there are indirect vestibulo-reticulo-spinal pathways (10). At the same time, when the head is moved by either a perturbation or voluntarily, other cervical-projecting LVST neurons are activated, influencing alpha and gamma motoneurons to forelimb muscles (15, 16).

Evolution of the bony skull from the early hominids to present humans, as revealed by the fossil evidence, brought about a progressive movement of its center of gravity in the mid-sagittal plane (X–Z plane) to slightly in front of the occipital condyles (viz. the fulcrum), thereby reducing the jaw and neck muscular masses but more importantly benefiting the stability of the head and neck (17, 18). The heads of extant mammals, and most notably those of subhuman primates, are still relatively massive and are positioned above a multilinked (typically seven) cervical spine, the most complicated articular system of the body, and behave as an unstable inverted pendulum on top of a highly mobile neck under most conditions. The range of mobility at the different cervical joints, such as the skull to C_1_, C_1_ and C_2_, C_2_ to C_3_, and C_3_ to C_7_, varies. The movements of flexion and extension, lateral bending from the midline, and axial rotation from the midline of the head and neck are not entirely confined to single loci but distributed along the cervical axis (19–23). The distribution of synaptic fields of VS axons from specific and confined segments to a more uniform innervation along the cervical segments likely corresponds to the mechanical structure and constraints of the evolved primate head and neck. The importance of the VS pathways to vertebrate survival is reflected in its early development and myelination (24–29), and the hodologically defined vestibular populations conserved from early vertebrate evolution in distinct and mixed neuroepithelial segments: projections to the VOR circuit originate from neurons in the rostral to middle rhombomeres and the origins of the ipsi- and contralaterally projecting VS pathways are found in the middle to the more caudal rhombomeres (30–32). The behavioral and functional manifestations of early vestibular maturation can be seen when a foal almost immediately after birth rights itself in direct opposition to the force of gravity and starts to correct body sway. Lambert et al. (33) demonstrated in mice that the vestibular-mediated drive to the cervical motoneurons continues rapidly in the first weeks postnatal. These striking demands on motor performance are possible due to the established sensory signals from the otolith and canal hair cells delivered by the VS system to the relevant motor effectors (34, 35). It is reasonable to postulate that the ontogeny of motor behaviors associated with the VS system reflects survival pressures among the vertebrate species.

Goldberg and Fernández published their landmark investigations of functional properties of semicircular canal afferents in 1971 (36–38) and established the squirrel monkey as a valuable experimental model of vestibular structure and function. The binocular visual field of the squirrel monkey (about 146°) and human (about 140°) are comparable (39), making the squirrel monkey a model widely used in vision research (40). The average squirrel monkey is a small primate (~800 g), having a relatively high brain weight to body weight ratio (approx. 1:25 versus the human’s 1:50). Its encephalization quotient, the ratio of brain mass to body mass, of 2.8 places it the fourth highest among those of the fifty primate species studied by Jerison (41), and has proven to be a valuable model in clinical neuroscience (42, 43).

This study aimed to provide a structural foundation for subsequent functional studies in alert and behaviorally trained squirrel monkeys to specify the role of the VS pathways in neck-eye coordination in relation to gaze. The significance of head stabilization is seen in our ability to change gaze orientation, maintain binocular fixation during most activities, detect self-motion, and adaptively react to externally imposed head/body perturbations. Despite the importance, wide gaps remain in our understanding of basic VS mechanisms. The present study provides a descriptive analysis of the anatomical organization of identified secondary MVST neurons, VOC neurons, and cervical-projecting LVST neurons in the squirrel monkey. We used intracellular recording, dye labeling, and orthodromic and antidromic stimulation protocols to map and compare the projections and terminations of VS neurons in the brainstem and cervical spinal segments. The primary advantage of the squirrel monkey in this experiment is the relatively shorter length between its cervical segments (~2–3 mm) compared to that of the cat (5–12 mm, personal observations), thereby permitting the visualization of labeled axons, their branching patterns, and terminal synaptic fields within the brainstem and over a greater number of spinal segments. These techniques were also used to identify selected targets, such as central cervical nucleus neurons, sternocleidomastoid, trapezius, and splenius motoneurons, and map their connection to the VS pathways. The results in the primate support the seminal work done by Shinoda and his team in the cat (44–48), particularly the VS trajectories and branching distributions in the cervical spinal cord. The major findings of the study also extend those studies by including, where possible, VS synaptic projections to brainstem neuron pools, complete mapping of individual VS axons in the cervical segments, 8th nerve synaptic input(s) to likely targets of specific spinal neuronal populations, and the likely matching of VS innervation to the skeletomuscular organization of cervical spinal cord. Several review articles have partially described the findings (5, 9, 49, 50).

## Materials and methods

### Surgical preparation

All surgical and experimental procedures were conducted according to the National Institutes of Health Guide for the Care and Use of Laboratory Animals, 8th edition, and approved by the Institutional Animal Care and Use Committee at Oregon Health & Science University (OHSU), Portland, OR. All experimental procedures and tissue processing were conducted at OHSU.

Experiments were performed on adult male squirrel monkeys, *Saimiri sciureus* (NCBI: txid9521), weighing ~0.8–1 kg. All animals were initially anesthetized with an intramuscular injection of ketamine (40 mg/kg) and acepromazine maleate (1 mg/kg). A femoral vein was cannulated, and a continuous intravenous infusion of 11 mL lactated Ringer solution, 5–10 mg sodium pentobarbital, and 0.2 mg dexamethasone per hour was maintained. Systemic arterial pressure was continuously monitored using an indwelling catheter in the femoral artery, which was placed in series with a pressure transducer. Rectal temperature was kept near 38°C by a heating pad and radiant lamp. After surgical preparation, including tracheal intubation, the animal was immobilized with pancuronium bromide (100 μg/h) and ventilated with room air; end-tidal P_CO2_ was monitored and kept near 4%. A bilateral pneumothorax was done to reduce respiratory pulsations of the spinal cord to facilitate intracellular recordings; atelectasis was controlled by routine hyperinflation of the lungs.

### Neural recording and staining

**Figure 1:**
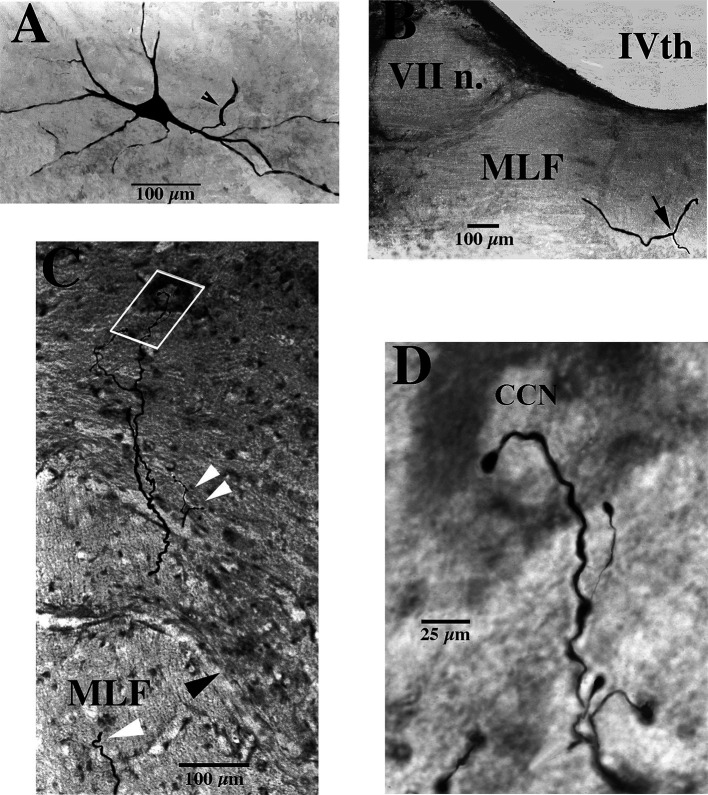
Soma, axon bifurcation, and terminal synaptic morphology of two VOC neurons. **(A,B)** Photomicrographs show the labeled soma of a VOC cell and the initial segment of its axon (arrowhead) recovered in the ventral Lateral Vestibular Nucleus in panel **(A)** and its axon bifurcation (arrow) in the contralateral MLF rostral to its soma in panel **(B)**. **(C,D)** A separate VOC neuron is shown penetrating the ventral horn of lamina VII dorsal to the medial wall of lamina VIII (indicated by the black arrowhead). The axon branches targeted cell groups in lamina VII (double white arrowheads) and a neuron (white box) in the central cervical nucleus (CCN) in panel **(C)**. The presumed targeted CCN neuron (located immediately dorsal to the labeled processes in this image; 60 μm horizontal section) is shown in the enlargement along with the terminal processes of the VOC collaterals in panel **(D)**. The neuron received short-latency, ~2 ms, disynaptic excitatory post-synaptic potentials from shocks applied to the contralateral vestibular nerve (Vc) that summated into action potentials. See Methods and Results for more details. Calibration bars are given in each panel.

**Figure 2:**
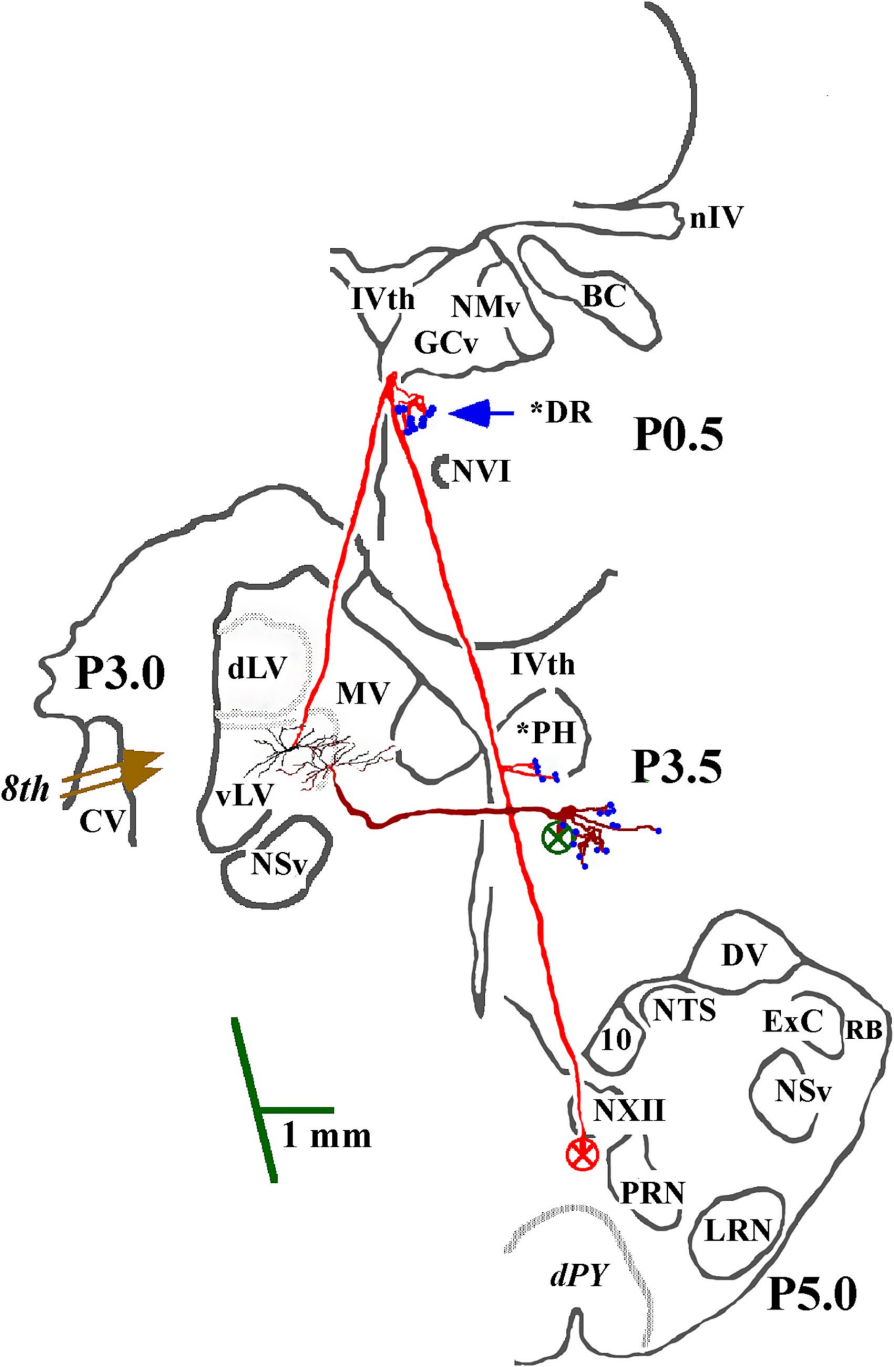
Soma location and initial axon trajectory and branching in the caudal brainstem of two cMVST neurons. Neurons were intrasomatically recorded and labeled to show their axon trajectories and innervation within the caudal brainstem, although limited. The axon of the more lateral neuron (in red) projected rostrally from its soma. It crossed into the contralateral MLF, like VOC neurons, but without bifurcating into ascending and descending branches, issued synaptic specializations in the dorsal Raphe (*DR), reversed course and projected caudally in the descending MLF with a minor input to the prepositus hypoglossal (*PH) nucleus. The other cMVST neuron projected across the midline into the descending MLF and issued collateral input to the PH nucleus. Abbreviations are given in Methods.

**Figure 3:**
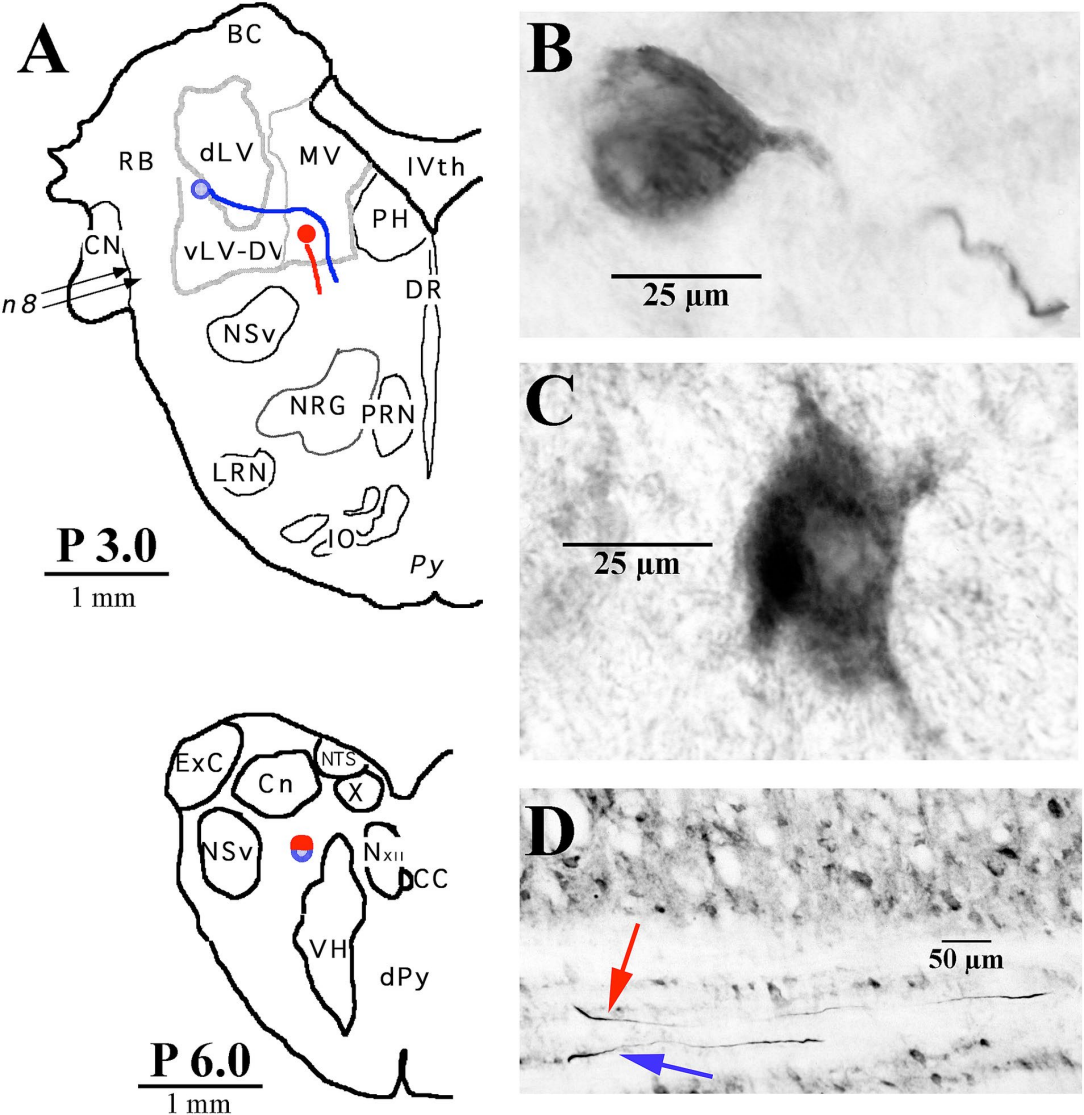
Two examples of LVST soma locations in the vestibular nuclei and their initial axon trajectories in the brainstem. **(A)** Soma labeled in blue was located in the ventral lateral vestibular nucleus (vLV) and is shown in the photomicrograph in panel **(B)**, and the other neuron labeled in red was located in the ventral portion of the medial vestibular (MV) nucleus and is shown in the photomicrograph in panel **(C)**. The lower panel **(A)** gives a schematic representation in the transverse plane of the two axons, traveling in tandem at P 6.0 in the caudal brainstem. **(D)** Photomicrograph of the two axons in the sagittal plane at P 6.0 coursing in a fiber track below the cuneate nucleus (Cn). Both neurons were intra-somatically labeled and antidromically activated from pulses applied at C_1_. Axons could not be followed into the spinal sections. Abbreviations are given in Methods.

Intracellular recordings were made from axons between C_1_ and C_3_ with glass micropipettes filled with 3–4% biocytin (Molecular Probes; Cat# B-1592) or 2.5% neurobiotin (Vector Labs; Cat# SP-1120) in 2 M NaCl, 0.05 M KCl, and 0.1 M Tris buffer (pH 7.4; R = 30–50 MΩ). After sufficient axon injections were completed, intracellular recordings were then made from cell bodies of cervical spinal neurons with glass micropipettes filled with 2–4% biocytin dissolved in 0.5 M KCl and 0.05 M Tris buffer or 2.5% neurobiotin in 1 M potassium methyl sulfate (CH_3_KO_4_S) and 0.05 M Tris buffer (pH 7.36). In several pilot experiments conducted at the end of a separate study (51), the somas of selected VS neurons, identified by their monosynaptic input from the 8th nerve and antidromic activation from pulses applied to an electrode array at C_1_ but not at C_6_, were labeled with biocytin as described above; the data from these experiments were unreported and served as a prelude to the present study. Examples are given in Figures 1A,B of a VOC neuron, in Figure 2 of two cMVST neurons, and in Figure 3 of two LVST neurons. Since the primary focus of the study was to describe the synaptic organization of VS axons in the cervical spinal cord, stimulating electrodes were not implanted at C_1_ unless specifically stated because the integrity of the cervical segment at the electrode site was potentially compromised.

For both axon and soma recordings, DC- and AC-coupled records were conventionally preamplified, filtered below the Nyquist frequency, and externally amplified to span the 12-bit range of the data acquisition device (1401Plus, Cambridge Electronic Design (CED), Cambridge, England) interfaced to a 80×86 computer. The cell’s AC- and DC-coupled voltages were sampled at 20 kHz and 3.5 kHz, respectively, in the acquisition software (Spike2, CED). In all cases, electrical pulses consisted of 100-μs constant-current shocks delivered via a stimulus isolation unit at 1–2/s as a single stimulus, as 3–5 pulses in a train, or in brief cathodal or anodal polarization of the 8th nerve electrodes and time stamped events. Data were transferred to a Macintosh platform and visualized later using scripts written for the Igor (WaveMetrics, Lake Oswego, OR, USA) package.

Impaled axons and cells initially had resting potentials of −50 to −70 mV. To stain the individual neurons with biocytin or neurobiotin, negative current was passed through the electrode to hyperpolarize the cell from −2 to −5 mV and positive current pulses of 8–25 nA, 1/s, 70% duty cycle were applied. During the injection phase, the membrane potential was monitored during the off-cycle, and the injection was periodically stopped to inspect the evoked potentials. An injection was stopped if the resting potential rose more positive than −20 mV. Only one injection, or attempted injection, of a cell was made in an individual electrode track, and the electrode was immediately withdrawn from the tissue. A minimum distance of 1 mm was kept between injection sites, and axons were sought on both sides of the spinal cord. *No* attempt was made to first extracellularly record spontaneous or induced discharges before penetrating the axon. This can give potentially false results. Based on the cell’s responses to applied stimuli from multiple sites, it was repeatedly observed that an axon extracellularly recorded first was not the same as that immediately penetrated, although one or more responses could have comparable latencies. Therefore, all identification and labeling procedures were done only under intracellular conditions.

### Electrophysiological identification of 8th nerve input

**Figure 4:**
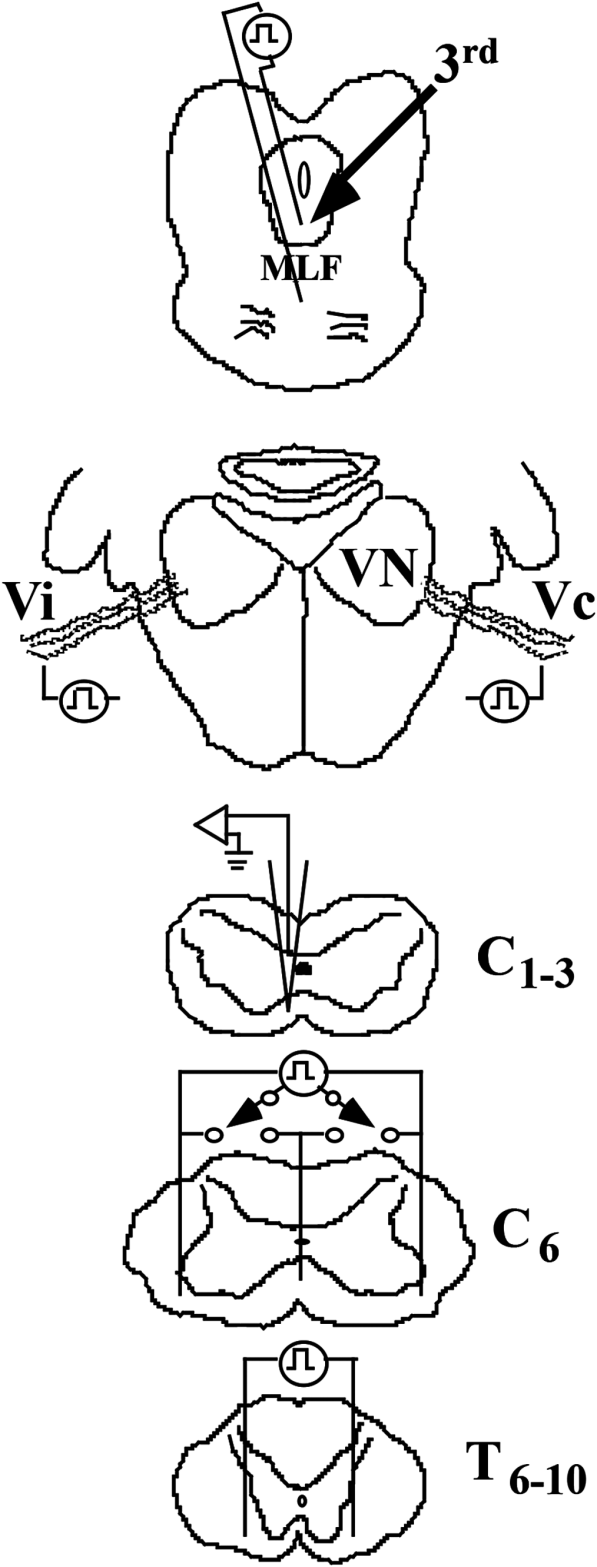
Methods to identify VS neurons in the squirrel monkey. Cartoons show the placement of stimulating electrodes: (1) in the rostral medial longitudinal fasciculus (MLF) at the caudal pole of the oculomotor (IIIrd) nuclei to identify a possible rostral ascending branch from a bifurcating axon, such as the vestibuloocular collic (VOC) neuron, (2) in the middle ear space to orthodromically excite ipsi- (Vi) and contralateral (Vc) 8th nerve afferents that project to the vestibular nuclei (VN), (3) in the ventrolateral funiculi of both sides and the centerline of the spinal cord at C_6_ to antidromically activate the descending axons that terminate in the lower cervical segments or pass more caudally, and (4) in the ventrolateral funiculi of both sides of the spinal cord at T_6-10_ to antidromically activate the descending axons of neurons that project to the lumbosacral spinal segments. Intra-axon and -soma recordings and dye labeling were made on either the left or right side and spaced at 1 mm intervals at C1-3.

Using a retroauricular approach to expose the middle ear on both sides, one insulated Ag/AgCl_2_ silver wire (250 μm dia.) bared for 1 mm at its tip (cathode) was inserted into the perilymphatic space of the vestibule through a hole bored in the promontory between the round and oval windows to electrically excite fibers in both the superior and inferior divisions of the 8th nerve; a bared coiled Ag/AgCl_2_ silver wire (anode) was placed rostrally in contact with the bone. Vestibular neurons were identified by their synaptic responses to bipolar pulses applied to the wires on the ipsilateral (Vi) or contralateral (Vc) 8th nerves (Figure 4). Secondary cervical-projecting vestibular neurons were selected for study based on two criteria: (1) the cell’s latent period of orthodromic response to electrical stimulation of the 8th nerve did not exceed 1.6 to 2.0 ms from C_1_ to C_3_, indicating a monosynaptic connection to the vestibular nerve afferents, and (2) the cell was not antidromically activated from the T_6-10_ stimulation site.

### Electrophysiological identification of cervical-projecting vestibular neurons

Figure 4 shows a schematic representation of the recording and stimulation techniques. Dorsal laminectomies were made to expose the spinal segments of C_1_–C_8_ and T_6-10_. Single axons and neurons were recorded between C_1-3_ on either the left or right side, and dye injections were spaced at 1 mm intervals and mapped to an individual drawing with landmarks. Dye spots were made at the end of the experiment to mark each cervical dorsal root entry bilaterally. The actual laterality of the recorded axon or neuron was verified by the dye spots, the specific mappings, and principally by histologically examining 60 μm horizontal serial sections.

At C_6_, an electrode array of 3 Ag/AgCl wires (200 μm dia.), insulated to within 1 mm of their tips, was placed in the ventrolateral funiculi on both sides, and the center wire was inserted along the midline to a depth beneath the central gray matter. Bipolar pulses were applied to combinations of the wires to estimate laterality for those axons projecting to that level of the spinal cord. Between T_6-10,_ two stimulating wires were inserted into the ventrolateral funiculi on either side to activate lumbosacral-projecting axons. Antidromic spikes were recognized by their constant latent periods for near-threshold shocks and by their collision with orthodromic action potentials evoked by 8th nerve stimulation or spikes evoked by intracellular depolarizing currents.

### Electrophysiological identification of ascending projection to the oculomotor nuclei

To identify the existence of a vestibular neuron having a bifurcating axon with both an ascending and a descending projection, a low-impedance glass microelectrode was lowered into the brain based on 3-D stereotaxic coordinates to target the rostral MLF between the IIIrd and IVth cranial nuclei (approximately A3.0; (52)). The optimal placement was guided by recording the field potentials evoked by Vi and Vc stimulation. The recording electrode was then replaced by a pair of insulated tungsten wires, separated from one another by ~3 mm and each bared for 1 mm (Figure 4); their placement was confirmed by recording comparable 8th nerve evoked field potentials with the stimulating electrodes. The location of the MLF electrodes was later confirmed in histological sections in each animal.

### Electrophysiological identification of spinal neurons

Motoneurons supplying the splenius capitis (SPL) and sternocleidomastoideus (SCM) muscles on both sides were identified by their antidromic response to electrical stimulation of their peripheral branches at C_2_–C_3_. Flexible bipolar stimulating cuffs were routinely implanted on one or more branches of each muscle. The SPL insertion to the occiput was left intact but detached from the spinous apophyses; the SCM was left intact but retracted laterally to expose the nerve branches. In a few experiments, the C_1_ dorsal rami were also prepared for activating the suboccipital nerve supplying the rectus capitis posterior major and minor and the obliquus capitis superior and inferior; no neurons were identified from these sites in early experiments, and thus, this procedure was discontinued. Other likely targets of VS innervation, most notably central cervical nucleus (CCN) and cervical long propriospinal neurons were also sought based on spinal coordinates and T_6-10_ antidromic responses, respectively.

### Histological procedures

The animal was monitored and kept under anesthesia for 4–8 h after the last injection of an identified spinal neuron. This was done to provide more time for label diffusion within the VS axon and less time for the label transport to leave the soma of the spinal neuron. During that time, the laminectomy was extended to the thoracic stimulating array, and the dorsal root entry zones at each cervical and thoracic segment on both sides were marked by specific cuts and ink, and measurements of the distance from the obex to the spinal stimulating electrodes were taken. After the survival period, the animal was given heparin sodium (1,000 units, i.v.) and perfused transcardially, first with 1 L of 0.9% saline and then with 1 L of a fixative solution containing 4% paraformaldehyde and 0.2% picric acid in 0.1 M phosphate buffer (pH 7.4). The midpoints of each cervical and thoracic dorsal root entry zone were marked with dye and cuts, and the brain and spinal cord were removed and stored overnight in a solution of 30% sucrose in 0.1 M phosphate-buffered saline (PBS) at 4°C. Three blocks of tissue were routinely processed: precollicular midbrain to obex, obex to T_1_, and T_2_ to T_6_. Frozen 60-μm serial sections in the sagittal (brain) and horizontal (spinal cord) planes were collected in cold 0.1 M phosphate buffer, incubated in Vectastain (Elite ABC kit, 1:50 dilution; Vector Laboratories) with 0.2% Triton X-100 for >4 h, transferred to a solution containing 0.05% diaminobenzidine and nickel ammonium sulfate (0.6%) for >1 h, and reacted by the addition of 0.003% H_2_O_2_ in PBS. In the two pilot studies, VS neurons antidromically identified to project to C_1_ were intrasomatically labeled, and the brainstem and spinal tissue blocks were sectioned in the transverse plane and processed as described above; sectioning the tissue block, particularly the spinal segments, in the transverse plane critically limited the accurate reconstruction of the axon trajectory and collateralization. All sections were mounted on gelatin-coated glass slides, lightly counterstained with a modified methylene blue and basic fuchsin stain, and cover-slipped. Sections were examined using a Leitz microscope, and individually labeled axons were reconstructed using a drawing tube (camera lucida) at ×16 or ×25 magnification. Terminal and *en passant* boutons were examined under ×40 and ×100 oil objectives to estimate as precisely as possible their number within the terminal field for each branch. Digital images were captured with a Leica DC500 camera; common darkroom operations such as brightness and contrast were enhanced as needed in Adobe® Photoshop®.

*Key to Abbreviations used in Figures:* 10th (vagus) nucleus; 12, 12th (Hypoglossal) nucleus; BC, Brachium Conjunctivum; C, cervical; CC, Central Canal; CCN, Central Cervical Nucleus; CV, ventral cochlear nucleus; dLV, dorsal Lateral Vestibular nucleus (Deiters’); DM, dorsal motor neurons of lamina IX; dPY, decussation of the Pyramidal Tracts; DR, dorsal raphe nucleus; DV, Dorsal nucleus of the Vagus; ExC, External Cuneate nucleus; IO, Inferior Olive; IVth, IVth (Fourth) Ventricle; GCv, substantial grisea centralis, pars ventralis; LRN, Lateral Reticular Nucleus; MLF, Medial Longitudinal Fasciculus; MV, Medial Vestibular nucleus; NMv, mesencephalic nucleus of the Vth (trigeminal) nerve; NSv, spinal trigeminal nucleus; NTS, Nucleus of the Tractus Solitarius; NVI, VIth (Abducens) nucleus; NXII, 12th (hypoglossal) nucleus; RO; PH, Prepositus Hypoglossi nucleus; PRN, Paramedian Reticular Nucleus; RB, Restiform Body (inferior cerebellar peduncle); Roller’s Nucleus; nIV, nerve of the IVth (trochlear) nucleus; SA, Spinal Accessory (XIth) nucleus; T, thoracic; Vc, contralateral Vestibular (8th) nerve; Vi, ipsilateral Vestibular (8th) nerve; VII n., VIIth (Facial) nerve; VH, Ventral Horn; vLV, ventral Lateral Vestibular nucleus; VM, ventral motor neurons of lamina IX; VN, Vestibular Nuclei; VII, lamina VII (Rexed); VIII, lamina VIII (Rexed); X, lamina X (Rexed).

## Results

### General classification of neurons

Vestibulospinal (VS) were typed as ipsi- (i) or contralateral (c)-projecting MVST neurons based on their axon location in the descending MLF of the upper cervical spinal segments in relation to their monosynaptic input from the 8th nerve. For example, an iMVST and a cMVST cell were monosynaptically activated from the left 8th nerve, and their axons were located in the left or right MLF in the cervical segments, respectively. VOC neurons were comparably typed as cMVST cells but were, in addition, antidromically activated from electrical pulses applied to the rostral MLF between the IVth (trochlear) and IIIrd (oculomotor) nuclei, revealing a bifurcation of its axon in the brainstem. LVST neurons were monosynaptically activated from the 8th nerve, and their axons generally travel in the lateral funiculus as they enter the spinal cord at C_1_ and progressively migrate to the ventrolateral and ventral funiculi as they projected to the lower cervical segments.

### General morphology of MVST and LVST neurons

**Figure 5:**
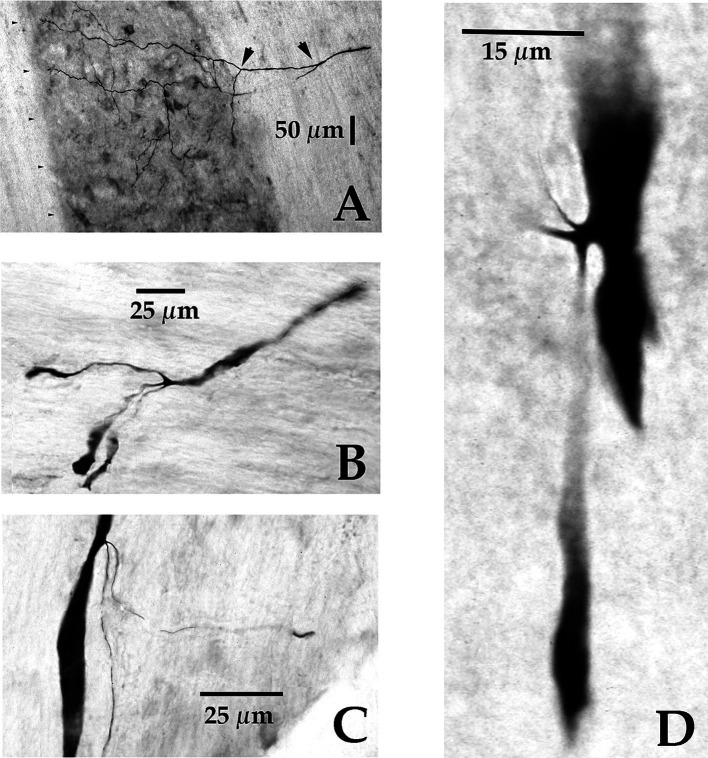
Axonal branching and collateralization of MVST neurons in the Cervical spinal cord. Photomicrographs show the typical branching features of two cMVST **(A,D)**, one VOC **(B)**, and one iMVST **(C)** axons projecting into the cervical spinal segments (taken from 60 μm horizontal sections). Calibration bars are given in each panel.

Four examples of branching from the parent MVST axon in the ventromedial funiculus are shown in Figure 5. Typically, two or more collaterals were issued from an axonal branch off the parent axon before the processes reached the medial wall of the ventral horn of laminae VII and VIII (examples shown in Panels A [arrowheads] and B); less commonly observed the branches were issued immediately off the parent axon and did not collateralize until they entered the ventral horn (Panels C and D).

**Figure 6:**
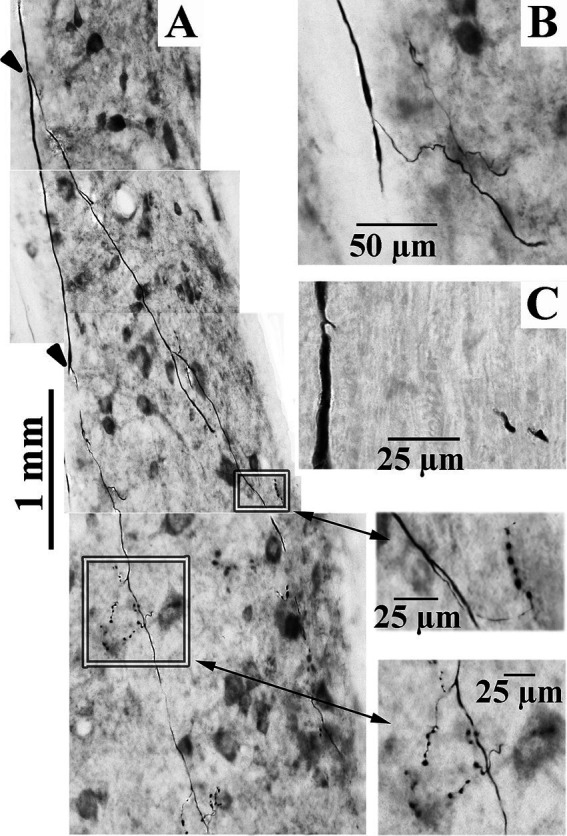
Axonal branching and collateralization of cervical-projecting LVST neurons. Photomicrographs show the typical branching features of three LVST axons innervating the cervical spinal cord **(A–C)**. Two branches [indicated by arrowheads in panel **(A)**] were issued from the parent axon in the lateral funiculus. Synaptic specializations from collaterals of these branches are highlighted by boxes and enlarged in the corresponding inserts indicated by double-headed arrows (taken from 60 μm horizontal sections). Calibration bars are given in each panel.

Three examples of branching from the parent LVST axon in the lateral funiculus at C_1_ are shown in Figures 6A–C. Typically, the first branches were issued near to or across the lateral wall of the ventral horn in lamina IX (Figures 6A,B); less commonly observed were collaterals issued from an axonal branch more distantly from the ventral horn (Figure 6C). All VS axons reported here were tested and found unresponsive to lower thoracic cord stimulation. Lumbosacral projecting LVST axons recorded and labeled in these experiments were reported in a separate study (53).

Of the 134 axons identified by their short-latency, monosynaptic input from the 8th nerves, 35 (26%) were successfully labeled to permit a moderate to complete reconstruction of their projection patterns in the cervical spinal segments. Of the 35 axons, 9 (25.7%) were classified as cMVST, 8 (22.9%) as iMVST, 10 (28.6%) as LVST, and 8 (22.9%) as VOC axons. The orthodromic latency from the 8th nerve stimulation averaged 1.37 ms ± 0.31 (SD; *n* = 35). Eight (4 cMVST, two iMVST, and two VOC) axons of the 35 (22.9%) received an inhibitory input from the opposite 8th nerve, and two (cMVST) received an excitatory input from both 8th nerves. The latency of antidromic activation from the rostral MLF for the VOC axons averaged 0.91 ms (±0.22 ms SD).

Table 1 gives the number of axon (parent) branches and synaptic (*en passant* and terminal) specializations validated for each group of VS neurons at eight different sites from the obex to the first cervical dorsal root entry at C_1_ to the most distal segment C_7_ to C_8_. Since the axons were recorded and labeled in the upper cervical sections, the given observations in the upper segments for each VS population are necessarily more complete (see Methods for criteria inclusion). The results at C_1_–C_2_ show that the VS population’s innervation density is not uniform. Between C_1_–C_2_, VOC and LVST exhibited the largest number of branches from the parent axon (44 total with a mean of 5.5 ± 3.2 and 43 total with a mean of 3.6 ± 1.9, respectively) and the highest number of observed boutons (5,068 total with a mean of 115 ± 126.5 and 5,088 total with a mean of 118 ± 107, respectively). LVST neurons maintained their dense innervation of the ventral horn from the obex to C_1_ and at C_2_–C_3_. The cMVST preferentially targeted the two middle cervical segments from C_2_ to C_4_, and iMVST cells, which are presumed inhibitory on their targets (54), exhibited a more sparse innervation of the cervical ventral horn.

**Table 1:** Morphological properties of secondary VS neurons in the squirrel monkey.

		VOC (*N* = 8)	cMVST (*N* = 9)	iMVST (*N* = 8)	LVST (*N* = 10)	All (*N* = 35)
Obex–C1	Br	22 (3.1 ± 2.5)	10 (1.7 ± 1.2)	5 (1.25 ± 0.5)	23 (2.6 ± 1.7)	60 (2.3 ± 1.8)
	Bou	2,320 (105.5 ± 100)	475 (47.5 ± 17)	808 (162 ± 142)	3,219 (140 ± 84)	6,822 (114 ± 94)
C1–C2	Br	44 (5.5 ± 3.2)	21 (3 ± 1.6)	14 (2.8 ± 1.9)	43 (3.6 ± 1.9)	122 (3.8 ± 2.4)
	Bou	5,068 (115 ± 126.5)	1,850 (88 ± 73)	874 (62 ± 33)	5,088 (118 ± 107)	12,880 (106 ± 105)
C2–C3	Br	30 (5 ± 2.8)	27 (3 ± 1.3)	9 (1.5 ± 0.8)	41 (2.5 ± 2.4)	107 (3.1 ± 2.2)
	Bou	1,678 (56 ± 63)	3,254 (120.5 ± 84.5)	329 (41 ± 24)	4,930 (120 ± 136)	10,191 (96 ± 107)
C3–C4	Br	17 (2.8 ± 2.3)	21 (2.5 ± 1.4)	4 (1.3 ± 0.6)	24 (2 ± 0.7)	66 (2.3 ± 1.4)
	Bou	1,423 (85 ± 66)	2,246 (94 ± 51)	245 (61.3 ± 53)	1,498 (62 ± 58)	5,412 (78 ± 58)
C4–C5	Br	1 (1)	8 (2 ± 1.2)	2	9 (2.25 ± 1)	20 (2 ± 0.9)
	Bou	188	608 (76 ± 22.5)	52 (26 ± 11)	284 (32 ± 31)	1,132 (57 ± 45)
C5–C6	Br	3 (3)	9 (2.25 ± 1.9)	2	6 (1.5 ± 1)	20 (2 ± 1.3)
	Bou	176 (59 ± 26)	473 (53 ± 22)	133 (66.5 ± 17)	384 (64 ± 70)	1,166 (58 ± 40)
C6–C7	Br	0	5 (2.5 ± 0.7)	0	4 (2 ± 1.4)	9 (2.25 ± 1)
	Bou	0	33 (7 ± 6)	0	183 (44.5 ± 20)	216 (24 ± 25)
C7–C8	Br	1	1	0	0	2 (1 ± 1)
	Bou	12	8	0	0	20 (10 ± 3)
All	Br	118 (14.75 ± 4.8)	102 (9.3 ± 6.5)	36 (4.5 ± 3.3)	150 (12.5 ± 4)	406 (2.8 ± 2)
	Bou	10,865 (92 ± 100/Br)	8,947 (85 ± 67/Br)	2,441 (70 ± 69/Br)	15,586 (104 ± 106/Br)	37,839 (93 ± 93)

Axon branching and synaptic innervation in the cervical spinal cord at eight locations from the obex to C_8_ of the identified vestibuloocular collic cells (VOC; *n* = 8), medial vestibulospinal tract cells supplying the contralateral (cMVST; *n* = 9) or ipsilateral (iMVST; *n* = 8) cervical segments (re: 8th nerve input), and lateral vestibulospinal tract (LVST; *n* = 10) cells. The total number of branches (Br) and observed synaptic specializations (Bou or boutons) for each population of VS neurons at the eight spinal locations (left column) are given with the mean ± SD (in parentheses). The total branches and synaptic specializations of each VS population are given in the lowermost row and their total at each cervical location (right column).

### Morphology of vestibuloocular collic (VOC) axons

**Figure 7:**
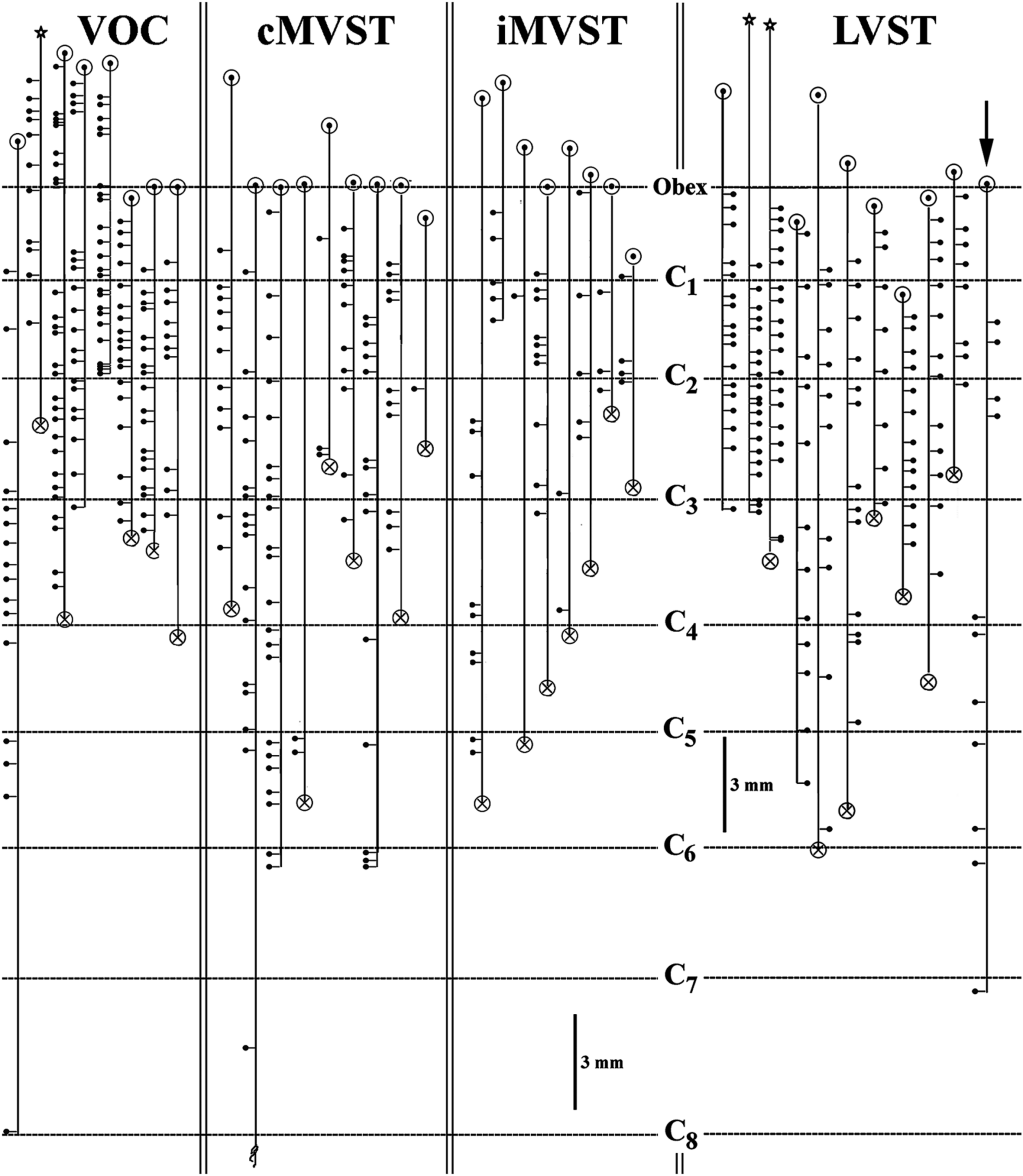
Branching summary for VOC (*n* = 8), cMVST (*n* = 9), iMVST (*n* = 8), and cervical-only LVST (*n* = 11) neurons. An open circle indicates the rostral extent of each recovered axon, and a circle filled with an X is the confirmed caudal location of the branch before the label fades. The star marks the location of the cell body of one VOC and two LVST neurons recovered from axon injections. The principal branch issued from the parent axon is schematically represented at each site and reflects the degree of specificity or uniformity of innervation. Three VOC, three cMVST, one iMVST, and four LVST neurons were followed to their termini. The arrow (at the far right side of the graph) designates the neuron presented in Figure 11B that showed characteristics of both LVST neurons in the upper segments and MVST neurons in the lower segments.

VOC neurons exhibited the more prolific innervation to caudal brainstem nuclei of the sampled population of VS neurons. In addition, VOC axons had expansive innervation patterns in the ventral horn of the cervical segments, mainly in laminae VII and VIII and, to a lesser extent, lateral and ventromedial lamina IX, from C_1_ to C_8_. Figure 1 shows four photomicrographs of two labeled VOC neurons. The VOC neuron shown in A and B was labeled intra-somatically (described in Methods): the soma and its axon (arrowhead) in the left ventral lateral vestibular (vLV) nucleus is shown in A and the same cell’s bifurcating axon (arrow) in the contralateral (right) MLF beneath the IVth ventricle about 1.5 mm rostral to its soma in B. The more dorsal branch projected rostrally and was antidromically-activated from pulses applied to the MLF at the level of the caudal IIIrd nuclei; the thinner branch projected to the cervical spinal cord, was antidromically activated by pulses to C_1_ electrodes used in this particular experiment but not activated by pulses to the C_6_ electrode array. Since the labeling of the descending branch faded at mid-C_1-2_ sections, it is not included in the Table, or the summary diagram of Figure 7. A separate VOC axon shown in Figures 1C,D was labeled intra-axonally at C_1_. The axon traveled in the right MLF (white arrowhead) and issued multiple collaterals that penetrated the ventral horn (black arrowhead) of lamina VIII and targeted cells with *en passant* and terminal boutons in lamina VII (double white arrowheads) and the central cervical nucleus (CCN); panel D is an enlargement of the area highlighted by the white box in panel C of the presumed terminal synaptic input to a CCN neuron.

**Figure 8:**
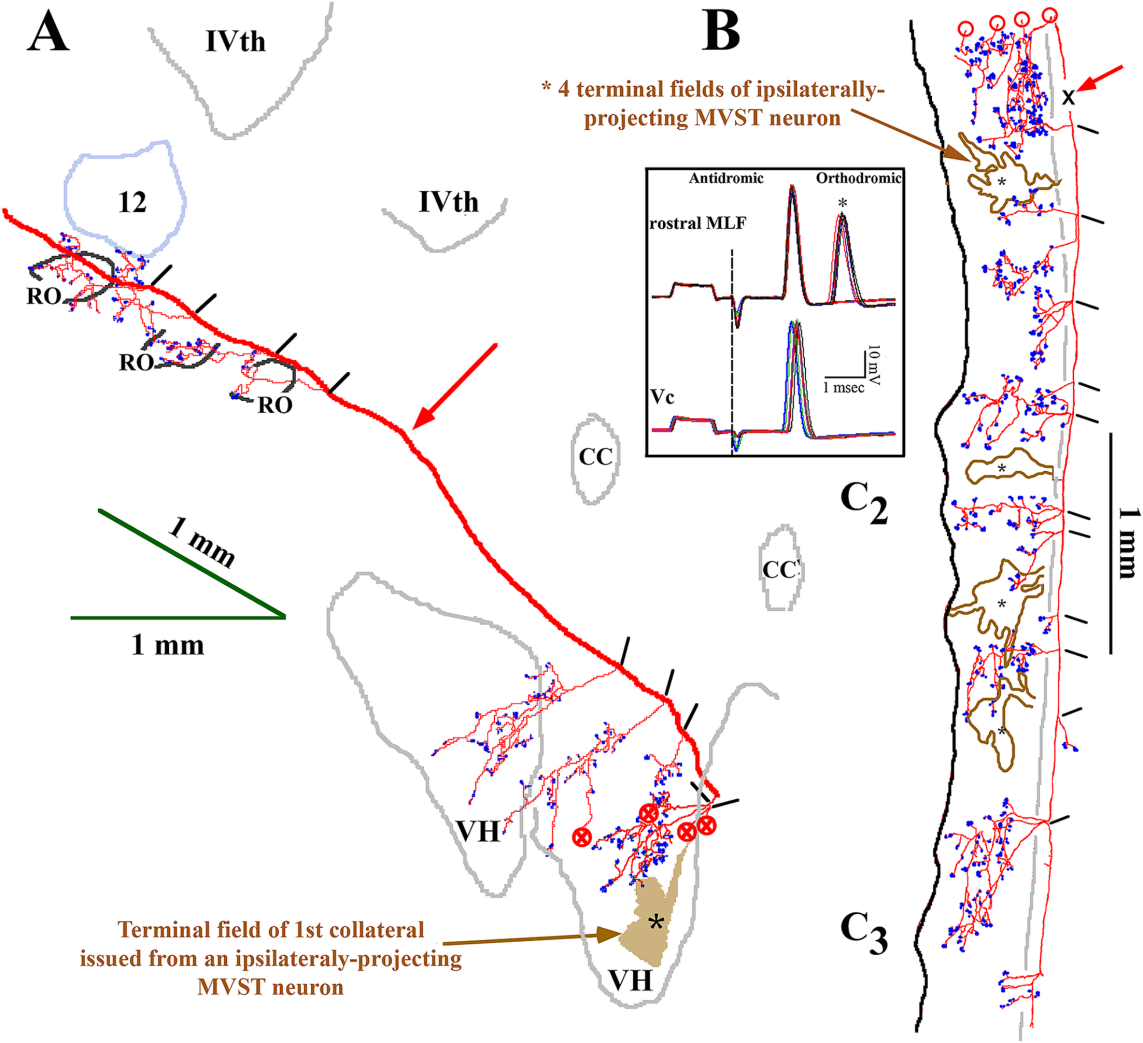
Extensive VOC (in red) and more confined iMVST neuron trajectories and synaptic innervation of the caudal brainstem **(A)** and cervical spinal cord **(B)**. Neurons were recorded, labeled in the same experiment, and shown together for comparison. Insert box shows the antidromic activation of the VOC neuron from stimulus pulses applied to the rostral MLF, followed by an orthodromic response to an unidentified descending input in the upper trace. The lower trace in the insert shows the monosynaptic excitation from the contralateral (Vc) 8th nerve (re: axon location in the spinal cord or ipsilateral to its soma). The descending axon of the VOC issued five collaterals caudal to its soma in the brainstem, four of which targeted the precerebellar perihypoglossal Roller’s nucleus (RO) and surrounding regions. As it coursed through the caudal brainstem [red arrow in panel **(A)**; sagittal sections] ventral to the IVth ventricle, no additional collaterals were observed until five collaterals were issued at <1 mm rostral to the obex that projected into the ventral horn (VH). The four collaterals continued caudally [marked by circles with an X in panel **(A)**] into the spinal cord (marked by open circles) in panel **(B)**. The red arrow in panel **(B)** marks the site (X) of the VOC recording and injection. The axon’s branching and innervation patterns were consistent along its course to its terminus at C_3_. The other MVST cell shown in the figure was monosynaptically activated from the left or Vi nerve. The iMVST cell issued its first recovered collateral at the junction of the obex and spinal cord and had four innervation fields [*shaded field in panel **(A)** and *open fields in panel **(B)**] into dorsal motor (DM) neurons of lamina IX and throughout laminae VII and VIII. The axon could not be followed caudal to mid-C_3_. Open circles in B indicate the processes project rostrally and are matched to their counterparts marked by the circles filled with an X in panel **(A)**. Calibration bars are given in each panel.

Complete innervation patterns of a VOC axon in the caudal brainstem (panel A) and cervical segments (panel B) are shown in Figure 8. The axon was recorded and labeled in the left ventromedial funiculus at C_1_ (marked by X and red arrow in B). The insert in panel B gives the cell’s identifying characteristics: the cell was antidromically activated at a latency of 1.25 ms, followed by a ~2.3 ms latency orthodromic response to shocks applied to the rostral MLF and received a monosynaptic input from the right or Vc nerve (re: axon location in the spinal cord) at a latency of 1.4 ms at threshold (12 μA) to a minimum latency of 1.2 ms (ca. 30 μA); the cell lacked any commissural synaptic drive from the left or Vi nerve or activation from C_6_ electrodes. In the caudal brainstem rostral to the obex (panel A; sagittal sections), the VOC axon issued four branches over a span of 1 mm to target Roller’s nucleus (RO), a precerebellar perihypoglossal nucleus (55, 56), and five more branches, again issued just rostral to the obex, that targeted the medial wall of the ventral horn (VH) near the sensory nucleus of V and the spinal V nucleus (substantia gelatinous of Rolando); four of these branches issued collaterals that continued more caudally and terminated near the C_1_ dorsal root entry zone (B, open circles; horizontal sections). In the cervical segments (panel B), the axon remained in the ventromedial funiculus and issued 11 branches over a 4.3 mm course before its terminus (with three minor collaterals) at C_3_. All branches issued off the axon and their associated collaterals projected into the medial wall of lamina VIII and had extensive arborizations in the CCN and across lamina VII often reaching the lateral border of lamina VII. An estimated 85 boutons were located in Roller’s nucleus, and 5,380 boutons were counted in its cervical projections.

**Figure 9:**
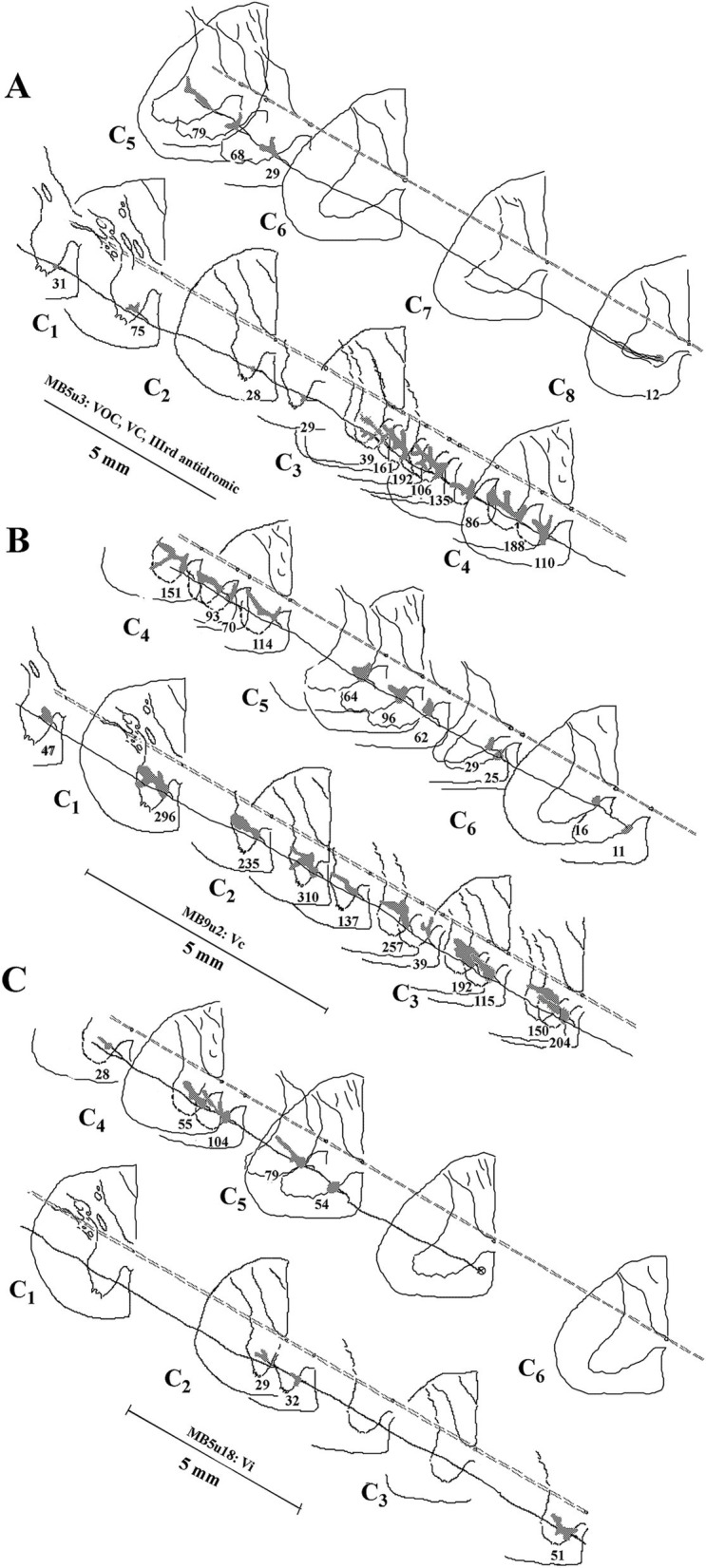
Comparison of the branching patterns and terminal fields of individual VOC, cMVST, and iMSVT neurons in the cervical spinal cord. **(A)** The VOC cell was recovered to its terminus at C_8_. The axon issued 15 branches that collateralized extensively to innervate the dorsal motor neurons of lamina IX, the lateral motoneuronal pool of lamina IX, and throughout laminae VII and VIII, particularly at C_3_–C_4_. **(B)** cMVST neuron issued 21 intra-spinal branches along its course to its terminus at mid-C_6_. Its innervation pattern was similar to that of the VOC cell in A, except its collaterals were more uniformly distributed along its trajectory. **(C)** iMVST neuron showed more restricted innervation zones that targeted mainly the medial wall of segments at C_4_–C_5_. The iMVST axon was recovered to the mid-C_5_ segment. Reconstructions were made initially from horizontal sections and transcribed into the transverse plane. The observed number of synaptic specializations within the terminal field is given for each branch of the individual axons.

The terminal synaptic fields of a C_8_-projecting VOC axon are presented in Figure 9A. The cell’s axon trajectory, branching pattern, and volume of each innervation field were reconstructed in the transverse plane from horizontal sections to better visualize its projection to the ventral horn. The number below each branch is the (estimated under ×40 and ×100 oil immersion objectives) number of boutons counted in each field. The cell exhibited 15 intra-spinal branches to its terminus at C_8_. Unlike the VOC axon described in Figure 8 that evenly targeted equivalent cell groups along its course, this VOC displayed a preference to target cell groups in C_3_–C_5_, with negligible projection into the ventral horn of C_1_ and C_2_ and avoiding C_6_ and C_7_ altogether. The 4 terminal fields at C_1_ and C_2_ had an estimated sparse 163 boutons, whereas the remaining 11 terminal fields in C_3_- C_5_ had an estimated 1,193 boutons. Nevertheless, the branching distributions to laminae VII and VIII by VOC axons from C_3_ to C_5_ were comparable to the VOC cell of Figure 8.

The branching summary of the eight VOC axons is schematically depicted in Figure 7 (VOC column). The labeled axons of four VOC neurons were sufficiently traced in the brainstem rostral to the obex and had extensive innervation to the perihypoglossal nuclei and the medial reticular formation. The label of three VOC axons did not extend rostral to the obex, and the remaining axon was recovered over only a brief extent rostral to the obex. Table 1 provides quantitative measures of the VOC axons (first cell column on the left). On average, each VOC axon issued 14.75 ± 4.8 (SD) branches in the cervical segments (total 118 branches); on average, 92 ± 100 (SD) boutons were found per branch with an estimated total of 10,865 boutons. The number of branches and the estimated number of boutons encountered from the obex to C_1_ and within the individual cervical segments of C_1_–C_8_ are given to highlight the projection and terminal distribution. Although valid comparisons along the cells’ trajectory are not possible because only 3 of the 8 VOC axons were completely reconstructed, whereas the remaining five were partially recovered, VOC branching and terminal distributions were heavily weighted to C_1_–C_3_ ventral horn and consistently targeted laminae VII, VIII, and to a lesser extent the motoneuronal pools of lateral and ventromedial lamina IX.

### Morphology of contralateral projecting MVST axons

The identifier distinguishing cMVST from VOC neurons was the absence of an ascending branch in the rostral MLF. Figure 2 gives the soma location, initial axon trajectory, and brainstem collateralization of 2 cMVST neurons intrasomatically recorded and labeled in the ventral portion of the lateral vestibular nucleus (vLV). The neurons were monosynaptically activated by pulses applied to the left 8th nerve, antidromically activated from pulses to the C_1_ stimulus array but not the C_6_ array, and did not respond to rostral MLF pulses. The axons were recovered in the vicinity of their somas but were not visualized in the spinal cord. Thus, they are not represented in Table or Figure 7. The axon of the more lateral neuron (in red) followed an ipsilateral rostral course ~2.5 mm to cross the midline beneath the IVth ventricle and immediately issued a collateral with an estimated 12 boutons into the dorsal Raphe nucleus (*DR). The axon then traveled in the contralateral descending MLF, issued a minor branch with an estimated six boutons into the prepositus hypoglossal nucleus (*PH) at the level of its soma, and continued without branching ~1.5 mm in the brainstem until the label faded. The other cMVST neuron, with a dendritic field in both the vLV and the medial vestibular (MV) nuclei, its axon projected medially and crossed the midline at ~0.5 mm caudal to its soma and immediately issued a relatively robust projection into the prepositus hypoglossal nucleus, and the label faded.

An example of the axon path, branching pattern(s), and innervation in the ventral horn of a cMVST axon is shown in Figure 9B. Its axon issued 21 intra-spinal branches from C_1_ to its terminus at C_6_ and densely innervated the medial wall of lamina VII and VIII and in about one-half of the cases the terminal field extended throughout lamina VII and supplied the lateral motoneuronal pool of lamina IX. An estimated 2,713 boutons were counted, ranging from 11 (terminus) to 310 boutons/branch (*N* = 22). Although the branches were relatively evenly distributed, more synaptic specializations were seen in the upper cervical segments of C_1_–C_4_.

The branching patterns of the nine cMVST axons are schematically represented in Figure 7. The labeled axons of only two cMVST neurons were traced into the brainstem rostral to the obex, and no branches were observed; however, as seen in Figure 2, cMVST axons did innervate cell groups in the more rostral brainstem. Like the VOC axon shown in Figure 9A, one cMVST axon provided consistent innervation to the ventral horn in the upper cervical segments and bypassed C_5_–C_7_ before it terminated at C_8_. Table 1 provides quantitative measures of the labeled cMVST axons. In total, 90 branches were located in the cervical segments, and each cMVST axon, on average, issued 10 ± 7 (SD) branches; an estimated 83 ± 68 (SD) boutons were found on average per branch with a total of 7,751 boutons. Six of the nine (67%) cMVST distributed equally weighted innervation to the cervical segments and consistently targeted laminae VII, VIII, and lateral lamina IX.

### Morphology of ipsilateral projecting MVST axons

Uncrossed iMVST axons represented the least dense VS input to the cervical spinal segments. Two examples of the innervation of iMVST axons are shown in Figures 8, 9C. In Figure 8, the iMVST axon was monosynaptically excited by shocks to the left or ipsilateral 8th nerve electrodes, did not respond to C_6_ pulse stimulation, and the recovered axon faded at mid-C_3_. In the same experiment, the iMVST cell was readily distinguishable from the VOC axon. It supplied a comparable synaptic distribution into laminae VII and VIII, extending into the lateral IX, and evenly provided an estimated 268 boutons among its five branches. Figure 9C shows an iMVST innervating mainly the mid-segments of the cervical cord with a relatively low number (432) of observed boutons in the terminal fields of its eight branches. Despite the extent of innervation in the cervical cord, the three groups of MVST axons, namely VOC, cMVST, and iMVST cells, target the equivalent spinal cell regions. The branch locations along the individual eight iMVST axons are given in Figure 7. Few branches were found rostral to the 1st cervical dorsal root entry zone, and branches were seen to either be tightly distributed to specific segmental cell groups or bypass multiple segments. Table 1 provides quantitative measures of the iMVST axons. In total, 36 branches were located in the cervical segments, and each iMVST axon, on average, issued 4.5 ± 3.3 (SD) branches; the number of branches per mm of recovered iMVST axon was statistically fewer than that for VOC axons (*p* < 0.005; ANOVA with Tukey post-hoc). An estimated 70 ± 69 (SD) boutons were found on average per iMVST branch with a total of 2,441 boutons; despite the variability among MVST axons, the number of boutons per branch was not statistically significant.

### Morphology of cervical-only projecting LVST axons

The somas of cervical-projecting LVST neurons co-mingle with MVST neurons in the vestibular nuclei, as revealed by electrophysiological and intracellular labeling techniques in the squirrel monkey (51). This was confirmed in several secondary LVST neurons intra-somatically labeled and shown in Figure 3. The two somas are schematically represented in panel A at Rexed P3.0: soma of the lateral neuron (blue symbol) is shown in the photomicrograph of B, and the soma of the medial neuron (red symbol) is shown in the photomicrograph of C. Their axons project through the ipsilateral caudal brainstem to the spinal cord as shown by drawing at Rexed P6.0 (Figure 3A). The photomicrograph in Figure 3D (60 μm sagittal section) shows the two axons in close proximity coursing in a fiber tract beneath the cuneate nucleus about 2 mm lateral to the midline of the brainstem rostral to the obex. The axons were not recovered in the spinal cord, and thus, they are not included in Figure 7. In contrast, at this location, the three classes of MVST neurons were found more medially (Figures 2, 9).

**Figure 10:**
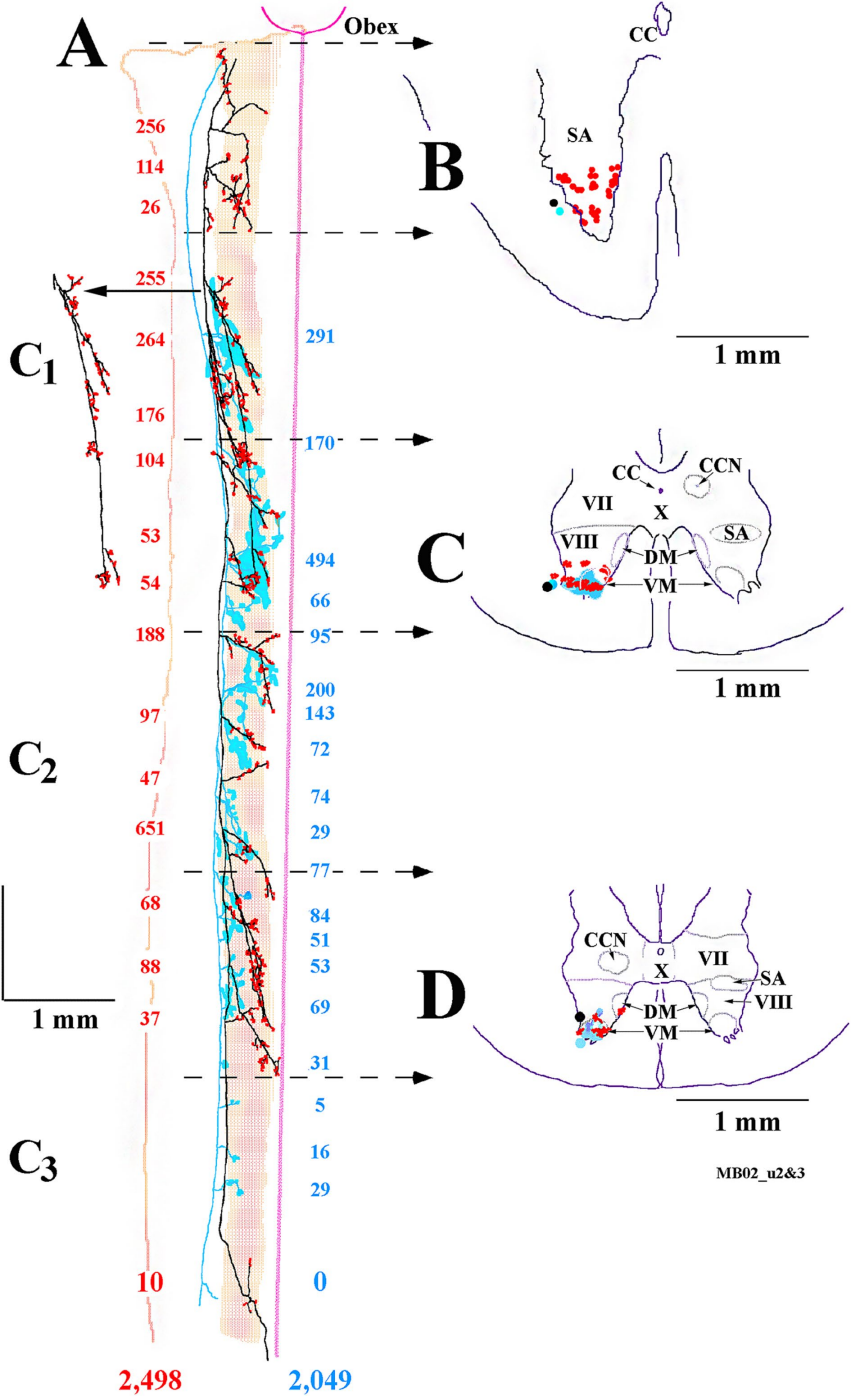
Prolific branching and overlapping terminal fields of 2 cervical-only LVST neurons to their termini at C_3_. **(A)** Reconstruction in the horizontal plane shows the parent axon and primary branches of one LVST neuron depicted in black and its synaptic specializations in red, and the other LVST axon and the extent of its synaptic innervation fields are drawn and outlined in blue. The number of synaptic boutons observed at each branch is color-coded and given for each axon. A reconstruction of a single collateral branch indicated by the arrow in panel **(A)** is added without modification to give better clarity. The midline from the obex to C3 is given as a straight line, the medial to lateral extent of the ventral horn is shaded, and the lateral edge of the white matter is drawn. Representations of each axon’s innervation to the ventral horn, transcribed to the transverse plane, from rostral to caudal, are drawn in panels **(B–D)**. Panel **(B)** is derived from the axon’s morphology from the obex to rostral C_1_ [obex to second dotted lines with arrowheads in panel **(A)**]. Panel **(C)** represents the spinal cord innervation at mid-C_1_ [between the third and fourth dotted lines with arrowheads in panel **(A)**]. Panel **(D)** represents the spinal cord at mid-C_2_ [between the fifth and sixth dotted lines with arrowheads in panel **(A)**]. Note both axons’ extensive, consistent, and overlapping innervation organization to the ventromedial motoneuronal lamina IX in the upper cervical spinal segments. Both axons were recorded and labeled in the same experiment.

**Figure 11:**
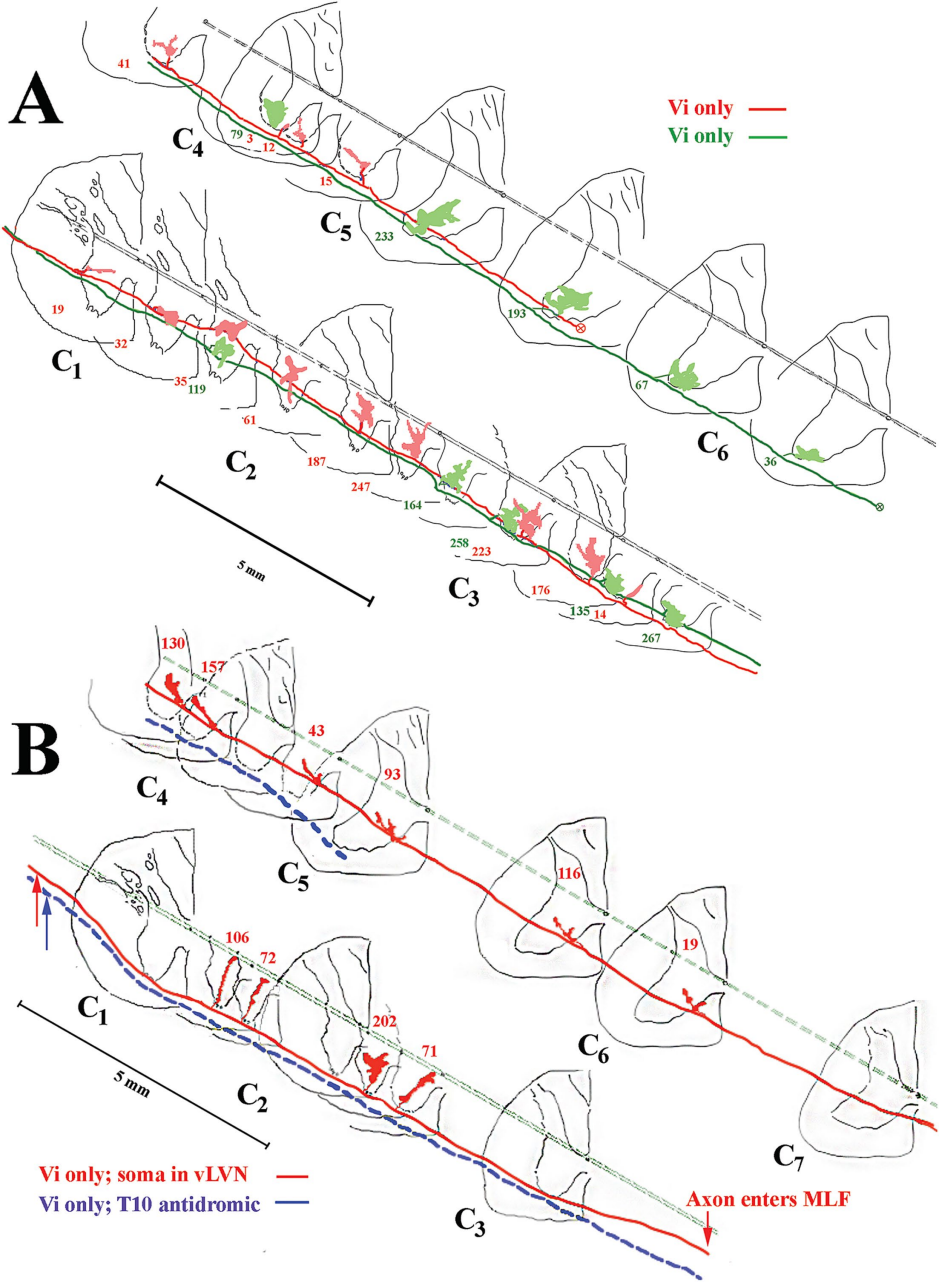
Common **(A)** and unique **(B)** projection and innervation patterns of LVST neurons to the cervical spinal cord. Data from horizontal sections were transcribed to transverse sections (same format as Figure 9). **(A)** Reconstructions of 2 cervical-only LVST neurons recorded and labeled in the same experiment. Both axons entered C_1_ in the lateral funiculus and slowly migrated into the ventrolateral funiculus as they traveled to mid-C_5_ or mid-C_6_ before the label faded. Both axons innervated comparable neuronal pools in lamina VIII and ventromedial lamina IX but targeted mostly separate segmental zones. **(B)** An exceptional VS neuron (labeled in red) behaved as an LVST counterpart between C_1_ and C_2_ but migrated from the ventral funiculus into the MLF between C_3_ and C_4_. The synaptic innervation changed to mimic the MVST counterparts in that tract and supplied cell groups in lamina VII and VIII. Another LVST neuron was antidromically activated by pulses applied to the lower thoracic spinal cord electrodes and labeled in blue. The injection sites of each axon are given with colored arrows. This LVST neuron did not issue a branch along its course before fading at C_5_ and maintained its position in the latero-ventrolateral funiculi comparable to other lumbosacral-projecting LVST neurons (53).

The extensive innervations of the upper cervical segments by two secondary LVST axons labeled in the same experiment are shown in Figure 10. Both axons entered the spinal cord in the ventrolateral funiculus and terminated at C_3_. The axon highlighted in black with red boutons issued 17 branches, and 2,498 boutons were estimated in its combined terminal fields; the axon sketched in blue issued 19 branches, and 2,049 boutons were observed (Figure 10A). Both axons had about two branches per mm of axon length. Panels B–D in Figure 10 provide transverse perspectives of the axon locations and their synaptic fields at 3 locations: caudal to the obex (B), in mid-C_1_ (C), and mid-C_2_ (D). In Figure 10B, the first 3–5 branches of one axon targeted the ventral regions and motoneurons of the spinal accessory nerve. In Figures 10C,D, both axons heavily targeted the ventromedial (VM) motoneuron pools of lamina IX, and to a lesser extent, the ventral regions of lamina VIII and the dorsomedial (DM) motoneurons of lamina IX heavily innervated by MVST and VOC axons. Figure 11A shows another set of 2 secondary LVST axons entering the spinal cord in the lateral funiculus. As the axons traveled in the lateral funiculus, branches were issued that projected medially into the central and medial zones of C_1_–C_3_. The axons migrated more ventrally along their paths, and the branches were directed more dorsally and into zones overlapping MVST axons. As seen in the branching summary for LVST axons in Figure 7 and the measures in Table 1, this abundance of branching in the upper cervical segments was a common feature of LVST axons. Although the sample is low, axon branching and synaptic innervation were not seen in the caudal brainstem (Figure 3) from separate soma labeling and rostral to the obex in axon labeling samples (Figure 7).

### Unique projection and innervation patterns of VS axons

An exceptional innervation of the spinal segments correlated with axon location was observed in one experiment and shown in Figure 11B. Two axons are shown, one in blue that entered the spinal cord in the ventrolateral funiculus and stayed its course until the label faded. This secondary neuron was antidromically activated from the lower T_10_ segment and displayed no branches in the recovered portion of its axon in the cervical segments; the lack of branching and synaptic distribution to the cervical segments of secondary lumbosacral projecting LVST in the squirrel monkey was documented (53). The other axon depicted in red also entered the spinal cord in the ventrolateral funiculus and issued four branches between C_1_ and C_3_ with terminal fields comparable to the other LVST axons. Interestingly, before the C_4_ dorsal root entry zone, the axon migrated out of the ventral funiculus and entered the ventromedial funiculus, the principal pathway of MVST axons, and coursed in this track location until its terminus at C_7_. Between C_4_ and C_6_, the axon issued six branches into the medial wall of lamina VIII and penetrated lamina VII, comparable to the three groups of MVST axons.

### Morphology and electrophysiology of spinal targets of VS axons

**Figure 12:**
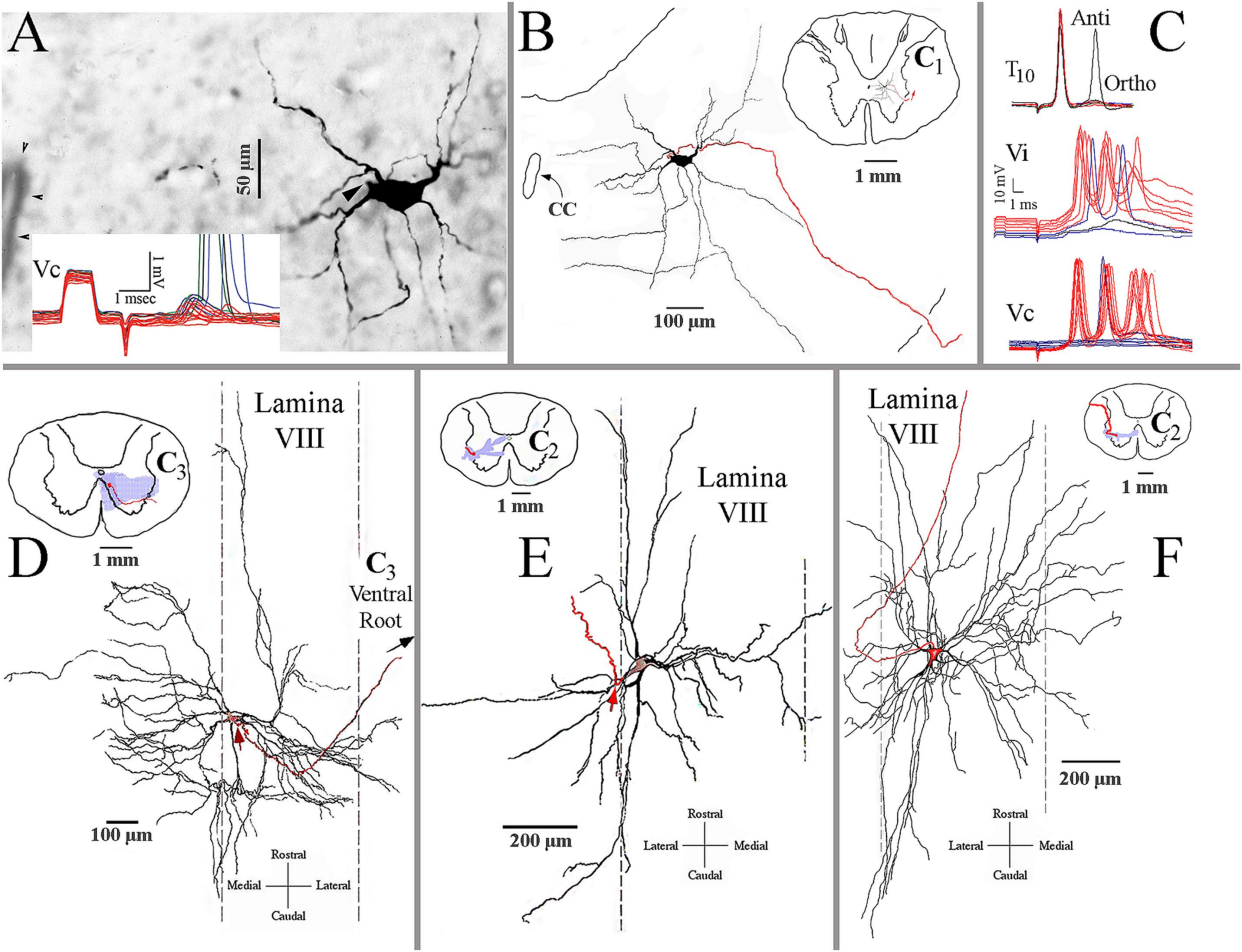
Identification and morphological properties of spinal ventral horn target neurons of VS axons. **(A)** Photomicrograph of a labeled central cervical nucleus (CCN) cell. Arrowhead marks the axon hillock. Insert shows the cell’s sub- and supra-threshold excitatory potentials from pulses applied to the contralateral 8th nerve (Vc). **(B)** Reconstruction of the CCN cell in A. The inserted drawing is a reduced spinal cord magnification at the CCN cell’s location. It shows the cell’s dendritic field is primarily within the nucleus and lamina VII. The cell’s axon is in red. **(C)** Presumed C_3_ long propriospinal neuron. The upper trace shows its short-latency antidromic activation (Anti) to pulses applied to the lower spinal cord at T_10_ and a longer latency orthodromic (Ortho) response from unidentified ascending input(s). The cell received bilateral and powerful synaptic drives from the ipsi- (Vi; middle trace) and contralateral (Vc; lower trace) 8th nerves. **(D)** Antidromically identified splenius capitis motoneuron recorded and labeled at C_3_ along the medial wall of lamina VIII. The motoneuron received both excitation and inhibition at disynaptic latencies from stimulus pulses applied to the contralateral and ipsilateral 8th nerves, respectively. The reconstruction is made in the horizontal plane and shows the expansive rostrocaudal, and particularly mediolateral, dendritic in the ventral horn. The red arrow indicates the location of the axon (in red) emanating from the soma. Insert is a schematic representation of the motoneuron’s soma and axon (in red) and its dendritic field (in shaded blue) in the transverse plane. Note the dendritic projections into the MLF medially and into lamina VII and X dorsally. **(E)** Antidromically identified sternocleidomastoid (SCM) motoneuron recorded and labeled at C_2_ along the lateral wall of lamina VIII. Same format as panel **(D)**. The dendritic field was mainly in lamina VIII but extended into lamina VII and X near the central canal. **(F)** Another antidromically identified SCM motoneuron was recorded and labeled at C_2_ along the lateral wall of lamina VIII. Same format as panel **(D)**. This SCM cell’s dendritic field was more extensive and confined medio-laterally and rostro-caudally in the horizontal plane within lamina VIII.

Electrophysiological identification and intrasomatic labeling were performed in the same animal to highlight the VS synaptic drive to neurons in the cervical spinal segments. A preferred target of squirrel monkey MVST axons was the central cervical nucleus (CCN). The CCN neurons link motor behavior from muscle spindles and joints to the brainstem and cerebellar neurons (57, 58). Figure 12 shows a photomicrograph of a labeled CCN neuron (A) and its C_1_ reconstruction (B). The insert in Figure 12A gives sub-threshold excitatory postsynaptic potentials (EPSPs) elicited from stimulation of the contralateral 8th nerve (Vc) that provoked supra-threshold action potentials as the stimulus intensity was increased. The soma and its dendritic tree were nearly two-dimensional in the transverse plane; its axon projected to the lateral funiculus and ascended. The cMVST and VOC, and to a lesser extent, iMVST axons innervate to CCN.

Another common target of likely all groups of VS axons is the presumed C_3_–C_5_ long propriospinal neurons. Figure 12C shows the electrophysiological characteristics of this neuron. The neuron was recorded in the central ventral horn and was antidromically (Anti) activated from T_10_ and received a longer latency orthodromic (Ortho) excitation from that site (upper traces in Figure 12C). Both ipsi-(Vi, middle traces) and contralateral (Vc, lower traces) stimulation of the 8th nerve elicited a powerful synaptic drive (multiple bursts of action potentials) to this cell at near supra-threshold shock strength. Although many attempts were made to label these neurons, no cell was sufficiently labeled.

MVST and VOC cells target the medial wall of lamina VIII, particularly at C_3_–C_4_. In this region, motoneurons innervate the splenius capitis muscle (59, 60). Figure 12D shows the two-dimensional reconstruction of an antidromically identified labeled splenius motoneuron in the horizontal plane. The motoneuron received disynaptic EPSPs and inhibitory postsynaptic potentials (IPSPs) from the contralateral and ipsilateral 8th nerves, respectively, that would be expected for rotation and lateral flexion of the head and neck. The dendritic field of this motoneuron extended from near the midline to the lateral margins of laminae VII and IX within the terminal fields of both MVST and LVST axons.

Strong 8th nerve synaptic inputs were also observed on antidromically identified sternocleidomastoid (SCM) motoneurons. This large, two-headed muscle originates from the sternum and clavicle and inserts into the mastoid process of the occipital bone. Its activation varies from head rotation to the opposite side when acting singularly or flexion of the neck and head extension when excited bilaterally. SCM motoneurons were encountered from the central regions of the ventral horn caudal to the obex to C_3_. Two examples are shown in Figures 12E,F. Both motoneurons were antidromically identified and received low threshold (presumably Ia input) and short-latency EPSPs from the SCM nerve. The motoneuron in Figure 12E had a dendritic field that spanned lamina VII and received disynaptic (latency 1.85 ms) EPSPs from both 8th nerves, indicating a primary role in neck flexion and sternum elevation needed for deep inhalation. The motoneuron in Figure 12F had a more expansive but more two-dimensional dendritic field along the horizontal plane that spanned laminae VII and VIII and received disynaptic (latency 1.9 ms) EPSPs from the contralateral 8th nerve.

## Discussion

The VS tracts exert powerful excitatory and, to a lesser extent, inhibitory influences on postural tonus and reflex control of head and neck stability and movement by their innervation of extensor (antigravity) motoneurons and associate interneurons of the cervical spinal cord. Understanding the relationship between the vestibular nuclei and spinal motor circuits is critical in interpreting the equilibrium mechanisms and the consequences of vestibular dysfunction in trauma and disease. The anatomical properties of a selected population of individual VS axons of the squirrel monkey identified by their direct connectivity to the vestibular nerve afferents and their destination in the brain and cervical spinal cord were presented in this study and will be discussed below.

The pioneering studies of Flourens (61) provided the foundation for the role of the labyrinthine structures in balance and reflex movement of the head and neck. Influenced by this work in the pigeon, the physicist Ernst Mach (62–64) analyzed the non-acoustic derived sensations of the inner ear and demonstrated a complete coordinate system for head rotations in three-dimensional space by the three semicircular canals. Studies by Alf Brodal and colleagues later confirmed the relationships of the vestibular nerve afferents to the brainstem vestibular nuclei (65), the vestibular nuclei to the oculomotor complex (66), and spinal cord (67, 68).

Anatomical tracing protocols developed from silver impregnation and regeneration methods [see (69), for an excellent review] to more precise techniques, such as autoradiography (70, 71), retrograde labeling (72–74), and the anterograde tracing (75–79), and so too did our knowledge improved of these descending vestibular pathways. The advent of the intracellular recording and dye labeling technique brought even greater precision and details of the neuronal linkage between the inner ear sensors to the head and neck spinal motor circuits.

One major advantage of the squirrel monkey model over the cat is the ability to track an individual VS axon from the caudal brainstem to its terminus in the cervical spinal cord. Two main VS populations were found (see Figure 7): those neurons that consistently innervated spinal targets across multiple segments, such as VOC, LVST, and, to a lesser extent, cMVST neurons, and those that innervated confined or regional targets, such as iMVST and some cMVST neurons. More than 20 pairs of muscles control neck movements and connect the skull and cervical vertebrae and should girdle and occur to varying degrees across the entire column (19, 80). Although the musculoskeletal organization of the neck is complex (81), three broad muscle groups can be recognized: (1) the large muscles that span multiple segments or the entire length of the cervical column and connect the skull to the cervical column, namely the long dorsal neck muscles (splenius, semispinalis, and longissimus capitii), or the skull to the shoulder girdle, namely the sternocleidomastoid and trapezius muscles innervated by the spinal accessory nerve; (2) the intervertebral muscles (splenius, semispinalis, and longissimus cervici) that connect the multiple vertebrae, and (3) smaller suboccipital muscles that serve to support the head on neck and allow extension and rotation of the head (82, 83) and short muscles that link adjacent vertebrae (60).

**Figure 13:**
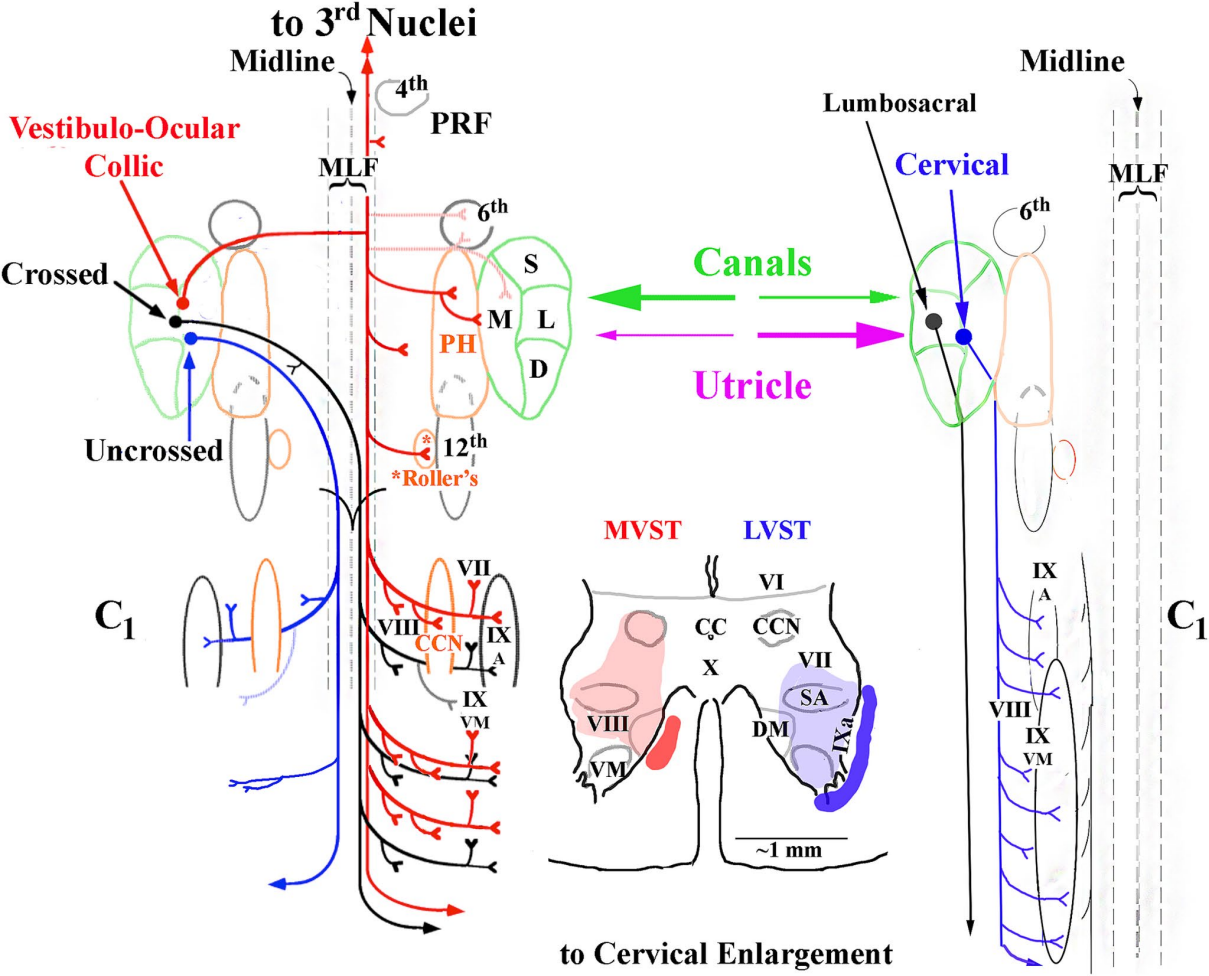
Summary of the major projections of crossed and uncrossed MVST (left side) and cervical-projecting LVST (right side) pathways to the upper spinal cord. The idealized organization of each pathway is viewed in the horizontal plane. Only neurons monosynaptically related to the 8th nerve to each pathway are represented. Vestibular inputs originate from the semicircular canals transducing angular head rotation and from the otolith organs (utricle and saccule), providing inertial acceleration cues and head orientation with respect to gravity into both pathways. These inputs can be from a single source, e.g., the anterior semicircular canal, or from multiple receptors, e.g., the horizontal semicircular canal and utricle, converging onto the neuron. The cartoon in the middle represents a single transverse section of the ventral horn (below lamina VI) to show an idealized axon location (solid) and synaptic terminal field (shaded) of MVST (red) and cervical-projecting LVST (blue) neurons. Secondary LVST axons projecting to the lumbosacral spinal segments bypass the cervical segments (see text for more details). Abbreviations used are: 3rd cranial nerve, oculomotor (medial, inferior and superior recti and inferior oblique extraocular muscles) nuclei; 4th, fourth cranial nerve or trochlear (superior oblique extraocular muscle) nucleus; PRF, pontine reticular formation; MLF, medial longitudinal fasciculus’ 6th cranial nerve, or Abducens (lateral rectus extraocular muscle) nucleus; S, superior vestibular nucleus; M, medial vestibular nucleus; L, lateral vestibular nucleus; D, descending vestibular nucleus; 12th, 12 cranial nerve or hypoglossal nucleus; CC, central canal of spinal cord; VI, VII, VIII, IX and X, gray matter layers of Rexed (156); CCN, central cervical nucleus; DM, dorsomedial motoneuronal pool; VM, dorsomedial motoneuronal pool; SA, spinal accessory motoneuronal pool supplying sternocleidomastoid and trapezius muscles [modified from (53)].

Figure 13 shows a summary representation of the major projections in the horizontal plane of the crossed cMVST and VOC neurons and the uncrossed iMVST (left diagram) and the uncrossed cervical-projecting LVST (right diagram) pathways in the squirrel monkey. The middle insert provides a generalized view of the axon location (solid) and innervation fields (shaded) of MVST (red) and LVST (blue) cells in the transverse plane of the spinal cord at C_1-3_. In general, the individual axons of both the crossed (contralateral 8th nerve activation) and uncrossed (ipsilateral 8th nerve activation) MVST axons in the primate course in the ventromedial funiculi (MLF) and innervate the medially located as well as the lateral motor pools, and their branching patterns resemble to a large extent those reported in the cat (45, 46). In this study, primate MVST axons were not seen to penetrate the central gray dorsal to lamina VII, and their synaptic innervation of the ventromedial motoneurons was less extensive than in the cat (47); cf. (76). Cervical-projecting LVST axons provide the more extensive innervation to the motoneurons of ventromedial IX in the squirrel monkey (see Table 1). In a broad sense, the MVST and LVST axons in both the cat (11, 12, 44–48, 84, 85) and primate appear to be distinctly separate in their trajectory and innervation patterns to motor pools in the lower cervical spinal cord. The following are the principal spinal ventral horn laminae targeted by both crossed and uncrossed MVST axons in the squirrel monkey. *First*, the medial wall of lamina VIII (Figures 5A,B, 8B, 9A–C), which contains motoneurons innervating the four dorsal suboccipital muscles (more rostrally) and intervertebral muscles (more caudally), the long dorsal neck motoneurons (Figure 12D), and interneurons including commissural cells (86, 87). *Second*, the neuronal pools of lamina VII: (i) local interneuron circuits, (ii) the central cervical nucleus (CCN; Figures 12A,B) which receives neck proprioceptive inputs from muscle spindles and joint receptors (57, 88) and projects to the cerebellar cortex, deep cerebellar nuclei, and brainstem (58, 89, 90), (iii) C_3_–C_5_ long propriospinal neurons [see Figure 12C; (91)] that participate in limb coordination and locomotion (92–94) across multiple joints (95), and (iv) scattered motoneurons supplying the ventral as well as the dorsal neck muscles. *Third*, the lateral motoneuronal groups of lamina IX (Figure 8B; Figures 9A–C) and particularly the spinal accessory (SA) neurons supplying the sternocleidomastoid (Figures 12E,F) and trapezius muscles that rotate the head and both tilt (unilateral activation) and flex (bilateral activation) the neck (96). *Fourth*, crossed MVST branching and synaptic terminal specializations were seen in the brainstem anterior to the obex (Figure 2). One such group is the midline dorsal nucleus of Raphe [DR; (97, 98)]; the importance of this target site is not clear, which has roles in various modulatory behaviors including wakefulness [see (99)]. Another brainstem target is the Nucleus Prepositus Hypoglossi (Figure 2). The importance of this cell group as a target is clear: the nucleus is essential for horizontal eye position (56), gaze [reviewed in Leigh and Zee (100)] and navigational (101) control. Crossed MVST axons display a high degree of collateralization in the cervical segments, contrasting with the fewer collaterals issued from uncrossed MVST fibers [examples are shown in Figures 7, 8B].

Vestibuloocular collic (VOC) neurons are a subclass of crossed MVST neurons. Ipsilateral-projecting VOC cells have been described in the cat (84), but only their contralateral-projecting counterparts in the squirrel monkey were encountered in the present study. VOC neurons possess axons that bifurcate in the contralateral MLF (Figure 1B), typically rostral to their cell bodies in the vestibular nuclei [e.g., Figure 1A], and target the oculomotor complex rostrally and the medullary reticular formation (102) (Figures 8A,B) and the cervical spinal ventral horn caudally, and thus are prime players in gaze (eyes in orbit plus head in space) control mechanisms. Their axon branching and synaptic boutons in the ventral horn were more extensive than the cMVST and iMVST samples (Table 1), and their termination fields resembled more closely those of other crossed MVST fibers (Figure 7). VOC cells also target the brainstem precerebellar Roller’s Nucleus (Figure 8A) in the caudal brainstem and the central cervical nucleus (Figures 1C,D, 9A) in the upper cervical segments. In contrast to the VOC neurons, the uncrossed MVST cells contacted these proprioceptive nuclei sparingly or not at all. These findings indicate that signals carried by VOC cells are widely distributed to include extraocular motor nuclei, the brainstem reticular formation, medially located cervical motoneurons, interneuronal segmental cervical circuits, such as the short- and long-projecting propriospinal neurons, and proprioceptive pathways to the cerebellum; and suggests broad and distributive roles of VOC cells in vestibular control of movement.

LVST axons travel through the ipsilateral brainstem caudal to their somas (Figures 3A–C), often together in fiber tracts in the ventral brainstem and into the ventral and ventrolateral funiculi of the cervical spinal cord (Figures 10A–D). These neurons branch extensively along their trajectory (Figure 7; Table 1), and target: (1) the alpha, gamma, and beta motoneurons in lamina IXa along the lateral wall, including the spinal accessory motoneurons; (2) the moto- and inter-neuronal pools of VII and VIII; (3) the motoneuronal pools of ventral lamina IX that innervate the neck muscles rostrally and the shoulder, proximal and distal limb motoneurons more caudally; and (4) often extending their terminal branches into the ventromedial cervical cord and thus overlapping with the synaptic fields of other MVST and VOC axons. Thus, the LVST neurons provide a dynamic and potent synaptic drive to the main players of neck posture and movements, such as the long dorsal neck muscles connecting the skull with the vertebral column (e.g., splenius, semispinalis, and longissimus capiti), intervertebral cervical muscles (e.g., splenius, semispinalis, and longissimus cervici), and the trapezius and sternocleidomastoid muscles activated by the spinal accessory nerve (XI cranial nerve) of C_1_–C_5_ (60, 103–105).

An unexpected observation is presented in Figure 11B that reveals an LVST axon can change its fiber tract [white matter plasticity, e.g., see (106)] as it courses through the cervical cord, in this case from the ventral funiculus to the ventromedial (MLF) funiculus. An antidromically identified lumbar-projecting LVST axon (blue dotted line in Figure 11B) traveled together with a separate axon (red solid line and terminal fields in Figure 11B) and maintained its position in the ventral funiculus without issuing a branch in the cervical spinal cord (until before fading at C_5_). This observation aligns with the results reported in a larger population of secondary lumbar-projecting LVST axons (53, 107). The axon’s innervation patterns reflected those of its neighbors at each site: in the upper cervical segments of C_1_–C_2_, the axon issued four branches into the ventral motor pools like other cervical-only projecting LVST axons (Figure 11A). The axon skipped the C_3_ segment, migrated into the ventromedial (MLF) funiculus at C_4_, issued six branches along its course to its terminus at C_7_, and its synaptic innervation patterns of the six branches were like its neighboring MVST axons. It is not clear what axon guidance cues were present to initiate the axon’s move out of one tract (repellent) and into another (attractant) or the prevalence (common or spurious) of this phenomenon (108–110). The importance of this finding lacks crucial pieces of evidence: what were the vestibular signals carried by the axon along its length, and did the signals trigger the axon’s switch in its trajectory and innervation pattern? Also, can we assume that the signals carried by the axon are simply the same at its soma and each branch, from the proximal ones to more distal and diffuse processes? The reliability of signal propagation from soma to axon might not be perfect. Examples of signal transmission variations are seen in invertebrates (111). Emerging evidence (112) suggests that the transmission from the cell soma to the distal terminals is also more complex in mammals, such as presynaptic impulse-dependent axon plasticity (113, 114), impulse initiation at distal sites (115), and preventive mechanisms at or near the axon’s nodes of Ranvier to prevent branch-point failures (116, 117).

The VS axons share and exclude many of the same synaptic target areas along the cervical spinal cord: the MVST axons innervate the more medial and dorsal regions of the ventral horn, whereas the LVST axons target the more lateral and ventral regions of the ventral horn. However, overlaps of innervation fields and the distribution of branches issued from the parent axon and their synaptic bouton fields in the cervical segments varied within and among the different groups of VS axons (see Figure 7 and Table 1). Some cMVST (e.g., Figure 9B) and cervical-only LVST (e.g., Figures 10, 11) axons showed a relatively regular innervation pattern along their course. Other VS axons, such as the iMVST cell in Figure 9C and the LVST (blue) cell in Figure 10A, showed selectivity in their targets, providing a dense innervation to one segment or several segments, e.g., the C_3_–C_4_ segments by the VOC axon in Figure 9A, or completing skipping one or more segments along their paths. The VOC and LVST axons generally showed uniformity of innervation, and the cMVST axons showed a mixture of regularity and specificity. The innervation of iMVST axons was less extensive than the other VS axons and selectively targeted the upper cervical segments. The possible targets of iMVST axons were the suboccipital muscles, namely the obliquus capitis superior and inferior and the rectus capitis posterior major and minor, that contribute to head and neck posture and head rotation and extension (118). It is reasonable to assume that the branching and innervation patterns might correspond to a global function, such as broad excitation of moto- and inter-neuronal pools (e.g., Figure 12C) or a limited function to affect specific targets. It is also likely that VS axons converge onto common motor pools to coactivate muscles acting across multiple joints. Although co-contraction does not generate a greater effective torque or endpoint force, it does contribute to mechanical stiffness at the joint(s) (119). Modulation of stiffness at a given joint can importantly reduce the effect of external perturbations (120–123), and alpha-gamma motoneuronal coactivation contributes to the vestibular reflexes of the neck (124) and limbs (125). This organization might be structured to ensure that VS pathways’ wide variety of control signals include those originating from the semicircular canals and otolith organs (126, 127).

### Signals carried by VS neurons in the squirrel monkey

Vestibular nerve afferents differ in their discharge regularity (spacing of interspike intervals) and dynamic response properties to head movement; as a result of this diversity, a broad spectrum of sensory signals are sent into the reflex pathways emanating from the vestibular nuclei [(36–38); reviewed by Cullen and Goldberg et al. (128, 129)]. It can be reasoned that the afferents’ diversity aligns with the diverse, dynamic elasto-viscous and inertial properties associated with movements of the eyes in the head (VOR), head on trunk (VCR), and extension of the limbs (VSR). An electrophysiological paradigm was developed by Goldberg et al. (130) to query the functional significance of the distribution of these diverse synaptic inputs to vestibular reflex pathways. Using this protocol, Boyle et al. (51) found that cervical-projecting MVST and LVST neurons received predominant inputs from the irregular afferents, presumably to facilitate the dynamic load requirements of the head and neck reflexes. In contrast, VOC neurons received their major synaptic input from the more velocity-encoding regular afferents. In short, the individual VS axons link the broad spectrum of vestibular sensory signals to the cervical moto- and interneuronal pools vital to maintaining head posture and initiating the multitude of head movements required in the behaving animal. Individual vestibular neurons can carry a single canal or otolith signal, convergent signals from several canals (131), or a convergent input from a canal and an otolith (132), as well as a more gaze-related processed signal combining the labyrinthine input with eye position in orbit or eye velocity, or both. Most vestibular neurons carry convergent signals (133–138). A point that cannot be resolved in this study is whether or not a correspondence exists between the VS cell’s responses to adequate stimuli, which can vary, and its axon morphology in the cervical spinal cord.

These afferent inputs to identified secondary VS neurons were confirmed in the alert squirrel monkey (139, 140). Secondary VS neurons respond to the velocity of ipsilateral whole-body rotation, type I response, in the yaw plane (with the head held stationary in space) and also respond to external translational accelerations along the Earth’s horizontal axis with sensitivities up to 500 imp/s/g and a modulation tightly tuned to the stimulus direction. In addition to the vestibular signals carried by VS axons, the discharge of some VS cells reflected the position or velocity of the eyes in orbit or both. Separate VS neurons were also encountered that had a disynaptic connection to the ipsilateral 8th nerve and displayed a type II (contralateral) response to yaw rotation and, in some cases, also carried eye position and velocity signals. Thus, the VS system to the cervical spinal cord in the squirrel monkey is equipped with both the anatomical organization and neuronal discharge behavior in response to head and eye movements to affect head stabilization and the compensatory reflex control of the neck muscles of the animal at rest and in motion. However, the VS drive is not invariable and can be dynamically modified by the behavioral context in which the motion is made. Volition is a key intervening variable that overrides the ongoing VS drive to the cervical motor circuits, eliminating the discharge modulation but not the background discharge rate, and the VS system remains able to quickly respond to external forces during self-generated head movements (141, 142). The VCR and proprioceptive stabilizing reflexes available visual motion cues (143) routinely interact in behaving animals during purposeful behavioral tasks. In humans, these neck reflexes can be voluntarily suppressed when needed (144), such as during free fall (145, 146) to effectively fix the head with respect to the trunk (147–149), or can even be influenced by an illusionary sensation of self-motion or vection (150).

Although the cat and primate have contributed detailed morphological properties of individual VS neurons, mouse models provide critical evidence of VS organization in the brainstem and spinal cord on a global scale. The strengths of the mouse models are: (1) similar to the advantage of the squirrel monkey over the cat, the smaller size of the mouse neuraxis permits a possible complete morphological description of VS neurons, and (2) the application(s) of novel and evolving identification techniques and genetic constructs. Antero- and retrograde tracers have been applied to map VS projections in mice (151, 152). Synaptic transmission in tonically active and glutamatergic Deiters’ neurons, the source of LVST axons to lumbosacral spinal segments, has been recently explored using electrophysiological techniques in mice by Stitt et al. (153). The advantage of the mouse model in revealing the depth and complexity of VS organization is shown by the ability to use established and validated techniques to track structural and functional development in embryos [e.g., (154, 155)] and perinatally (24). Lambert et al. (33) applied a multitude of techniques to identify the morphological connectivity and functional changes in MVST and LVST neurons occurring during postnatal maturation. Like the cat and squirrel monkeys, the mouse cervical spinal cord’s medial and lateral motor columns receive differential innervation from the ipsi- and contralateral MVST and LVST neurons. Neck and forelimb motoneurons were shown to receive direct, monosynaptic excitation, like in the squirrel monkey, and significant vestibular-mediated polysynaptic inputs. Interestingly, the robustness of this motor output increases within days of birth, conceivably aided by the maturation of myelinated axons. The mouse has coordinated head and eye movements but lacks a fovea. Also, the orbit convergence in mice is roughly half that of the squirrel monkey and human, effectively reducing its binocular visual field from around 140° in primates to 40° (39). Thus, visually guided behaviors and oculomotor strategies in mice and primates operate within dissimilar, in part, structural frameworks. To enhance our understanding of the correspondence of VS innervation properties to head and neck, limb, and ocular motor performance in behaviorally relevant contexts and its clinical relevance, a comparative approach is needed in related and disparate vertebrate species.

### Limitations of the present study

A disadvantage of intracellular recording is the sampling bias toward larger cells and axons. This bias is compounded by the additional procedure of dye labeling by injection after cell identification. Both the high electrode impedance and capacitance can change during the recording and, in turn, affect the strength and duration of applied current needed to inject an adequate amount of label. This limitation results in a loss or fading of the label over distance, which is evident in the present reconstructions. For a single track in the spinal cord, the electrode was routinely balanced using the bridge circuit of the preamplifier and replaced as needed. Despite these efforts, an inherent sampling bias and injection failures occur. The selection of the squirrel monkey as the experimental subject helped, in part, to minimize these limitations by visualizing an individual branching and termination morphology over a greater number of spinal segments, including a complete or near complete resolution in numerous neurons. Another limitation of the present study is that the data are necessarily descriptive. The wide variability in morphological characteristics within and across the VS groups rendered the application of statistical tools to parse the data and establish robust significant differences misleading. The only reliable conclusion from the present results is that the iMVST are distinctly less complex and more restricted in their innervation of spinal neuronal pools compared to the cMVST, VOC, and LVST neurons.

## Data Availability

The raw data supporting the conclusions of this article will be made available by the authors without undue reservation.
